# Fast Kinetic Response and Efficient Removal of Methyl Blue and Methyl Green Dyes by Functionalized Multiwall Carbon Nanotubes Powered with Iron Oxide Nanoparticles and *Citrus reticulata* Peel Extract

**DOI:** 10.3390/nano15080603

**Published:** 2025-04-14

**Authors:** Erich V. Manrique-Castillo, Mercedes del Pilar Marcos-Carrillo, Noemi-Raquel Checca-Huaman, Bruno L. D. Santos, Waldemar A. A. Macedo, César A. Barrero Meneses, Edson C. Passamani, Jean-Marc Greneche, Juan A. Ramos-Guivar

**Affiliations:** 1 Grupo de Investigación de Nanotecnología Aplicada para Biorremediación Ambiental, Energía, Biomedicina y Agricultura (NANOTECH), Facultad de Ciencias Físicas, Universidad Nacional Mayor de San Marcos, Lima 15081, Peru; 2 Centro Brasileiro de Pesquisas Físicas (CBPF), Rio de Janeiro 22290-180, Brazil; 3 Centro de Desenvolvimento da Tecnologia Nuclear–CDTN, Belo Horizonte 31270-901, Brazil; 4 Solid State Research Group, Faculty of Exact and Natural Sciences, University of Antioquia-UdeA, Medellín 050010, Colombia; 5 Departamento de Física, Universidade Federal do Espírito Santo, Vitória 29075-910, Brazil; 6 Institut des Molécules et Matériaux du Mans (IMMM UMR CNRS 6283), Le Mans Université, 72085 Le Mans Cedex 9, France

**Keywords:** *Citrus reticulata* peel residues, cyanidin, low-cost biosynthesis, multiwall carbon nanotubes, dyes removal, water bioremediation, adsorbent reuse, water cleaning strategies, magnetic nanoparticles

## Abstract

Maghemite nanoparticles (NPs) were successfully developed using phenolic-rich extracts (cyanidin) from *Citrus reticulata* peel residues. The 11 nm maghemite NPs, obtained at 3% *w*/*v* and at 353 K, presented the optimal synthesis conditions. To improve dye adsorption performance, the synergetic adsorption behavior between these 11 nm NPs and multiwall carbon nanotubes was demonstrated. Prior to the adsorption tests, the aging effect on NPs was carefully assessed using various analytical techniques, which clearly showed the magnetite–maghemite phase transition. However, this had no impact on the cyanidin coating or adsorption properties. A remarkable percentage removal of (93 ± 3)% for methylene blue and (84 ± 3)% for methylene green was achieved in short equilibrium times of 10 and 25 min, respectively, with an optimum pH value of 5.5. Reuse experiments revealed that 90% removal for both dyes was achieved between the second to seventh regeneration cycles. Organic loading during these cycles was effectively confirmed by X-ray photoelectron spectroscopy and magnetic measurements. Dye adsorption involves a two-step mechanism: (i) electrostatic adsorption by the negative surface groups of the adsorbent (isoelectric point of 5.2) and the dye cationic groups and (ii) π–π stacking interactions between the aromatic benzene rings of the dyes, the hexagonal skeleton of the multiwall carbon nanotubes, and the phenolic ring groups of the biosynthesized sample. These results suggest that the low-cost modified phenolic adsorbent can be successfully applied to dye removal from water with promising recycling properties.

## 1. Introduction

The problem of environmental pollution is one of the most important global challenges currently facing humanity, mainly due to the progressive increase in health problems in local societies and the significant damage caused to the global environment. Two sources of these issues are related to natural phenomena and human activities, such as urbanization, industrialization, mining, and prospecting [1]. It is clear that contaminated water must be considered an urgent priority in this respect, as it is the fundamental element of human survival. It should be stressed that its deterioration affects its physicochemical composition, biological aspects, quality, quantity, and availability [2]. As far as water contamination is concerned, the main pollutants are those associated with the manufacture of synthetic organic dyes, as well as those from the textile, tanning, and paper industries, which consume large amounts of water and, statistically, most of them tend to discharge considerable volumes of wastewater into aquatic ecosystems [3].

In the textile industry, over 60% of the used dyes are of the azoic type, i.e., they contain one or more azo groups in their structures. In inefficient dyeing processes, between 15 and 50% of the dye is discharged into wastewater, degrading the aesthetic quality of water bodies by increasing the biochemical and chemical oxygen demand and harming photosynthesis and reducing the light penetration in water, thus affecting the performance of algae and plants. Growing aquatic plants also enters the food chain and promotes toxicity to fish and other living organisms that can metabolize them in their bodies. Therefore, they become toxic intermediates, directly affecting the health of aquatic organisms and their predators [4]. Besides that, in some cases, this dye-contaminated water is officially applied in agriculture by farmers in developing countries, immediately causing a negative impact on soil quality and crop germination rates. Consequently, dye wastewater must be effectively treated using green technologies to avoid negative impacts on the environment, human health, and natural water resources.

In this context, many approaches using nanotechnologies focus on remediation technologies, in particular, magnetic remediation, where the adsorbent can be removed from the effluent, reducing its impact and improving the water’s final quality [5,6]. Therefore, the scientific community should engage more proactively to better understand the properties of nanomaterials associated with their chemical and physical properties, giving them the potential to play an extremely important role in advancing sustainability [7]. With the aim of integrating nanotechnologies with sustainability, ecological methods of synthesis of nanomaterials have been developed in recent years, giving rise to what is known as green synthesis, which uses clean, safe, profitable, and eco-friendly processes [8]. Green species are generally used in these syntheses, encompassing, for example, specific enzymes, proteins, amino acid groups, antioxidants, or chemical structures, and phenols from organic materials [9].

In different studies on biosynthesis, it has been observed that the source of phenolic compounds plays an important role in the properties of nanoparticles (NPs), including aspects such as phase, shape, and size [10,11]. Different compounds have already been used for this purpose, for instance, (i) an aqueous extract of the fruit of *Ficus carica*, in the synthesis of spherical maghemite NPs (γ-Fe_2_O_3_) of 4 to 6 nm [10] and (ii) ethanolic and aqueous extracts of leaves of *Phoenix dactylifera* L. in the synthesis of iron oxide NPs (Fe_x_O_y_-NP), resulting in spherical hematite (α-Fe_2_O_3_) and magnetite (Fe_3_O_4_) NPs with average particle size ranging between 35 and 44 nm [11]. These are two of the many examples of the starting phenolic compound influencing the characteristics of the obtained NPs, highlighting the variability in size and phase depending on the synthesis conditions. Consequently, additional researches are still required to contribute deeply on the understanding of how different extract matrices in green synthesis may affect the NPs nucleation processes. From an environmental point of view, plants extracts facilitate the reduction and precipitation of iron oxides, hence reducing the primary need for inorganic chemical reagents, such as ammonium hydroxide.

In the context presented above, the use of *Citrus reticulata* peel extract in the green synthesis of iron oxide NPs for environmental remediation was studied. This study had four clear objectives: (i) the preparation of *Citrus reticulata* peel extract obtained at various concentrations and their respective optical and vibrational characterization; (ii) the use of the polyphenol-rich extract (anthocyanins, mainly cyanidin) at various concentrations to tune the biosynthesis of magnetic NPs, from which the γ-Fe_2_O_3_ NPs with a mean particle size of 11 nm and MWCNTs exhibited remarkable removal properties for both dyes; (iii) detailed evaluation of the application of the obtained material for the removal of methylene blue (MB) and methyl green (MG) dyes in aqueous media; and (iv) physicochemical characterization after dye adsorption. The effects of time, pH, adsorbent dose, initial concentration, temperature, and recycling properties were systematically investigated. The material studied here were systematically characterized using multiple complementary analytical techniques, including X-ray diffraction (XRD), Transmission Electron Microscopy (TEM), Vibrating Sample Magnetometry (VSM), ^57^Fe Mössbauer spectrometry, and X-ray photoelectron spectroscopy (XPS). The results indicated remarkable removal efficiency for short contact times and easy removal of the dyes from the synthetic water with an external magnet. Finally, the adsorption mechanism was proposed by analyzing the second and seventh regeneration cycles of the recovered samples. A two-step mechanism was elucidated, mainly attributed to (i) electrostatic interactions, and (ii) π–π stacking interactions between phenolic, hydroxyl, carboxyl groups and aromatic benzene rings found in the adsorbent and dyes.

## 2. Materials and Methods

### 2.1. Materials

The synthesis used 5.2 g of iron sulfate heptahydrate (FeSO_4_•7H_2_O), 6 g of iron chloride (FeCl_3_), and 15 mL of ammonium hydroxide (NH_4_OH to 28% *v*/*v*). A total of 700 mg of multiwall carbon nanotubes (MWCNTs) were purchased from Chengdu Zhongke Times Nano Energy Tech Co., Ltd., Chengdu, China, with the following technical data sheet: external diameter, 20–30 nm; internal diameter, 5–10 nm; length, 10–30 µm; purity > 98%; and specific surface area > 110 m^2^/g and synthesized by chemical vapor deposition. Methylene blue (MB), whose chemical formula is C_16_H_18_ClN_3_S at 0.05% in H_2_O *w*/*v*, and methyl green (C_27_H_35_BrClN_3_ZnCl_2_, at 1% *w*/*v*) were both purchased from MERCK.

### 2.2. Extract Preparation

**Scheme 1:**
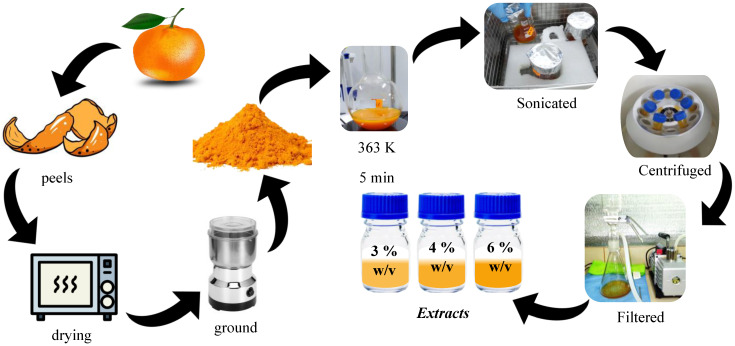
Procedure for obtaining *Citrus reticulata* peel extract.

*Citrus reticulata* peel was obtained by a process of washing, drying, and crushing as shown in Scheme 1. Specifically, 6 (3% *w*/*v*), 8 (4% *w*/*v*), and 12 g (6% *w*/*v*) of the dry mass were weighed; each mass was placed in 200 mL of hot water at 363 K for 5 min, cooled to 300 K in a water bath, dispersed in the sonicator, and centrifuged. It was then filtered with a vacuum pump, and the *Citrus reticulata* peel extract was obtained (see Scheme 1) and stored at 5 °C.

### 2.3. Biosynthesis

This process was carried out using mandarin peel extract at 3% *w*/*v* percentage (other high concentrations do not exhibit magnetic behavior) and by adding the ferrous salts. Specifically, it consisted of adding FeSO_4_•7H_2_O, FeCl_3_, and MWCNTs in an aqueous medium in a molar ratio of 1 to 2, dispersed for 10 min, and with the addition of 15 mL of NH_4_OH, from 5 mL to 5 mL until the dispersion reached a pH = 10. It was allowed to interact for 30 min, then cooled down to 300 K and washed until the pH was lowered to 7 (See Scheme S1). The sample was dried at 353 K for 10 h and labeled as NPM6.

### 2.4. Characterization

#### 2.4.1. Characterization of the *Citrus reticulata* Peel Extract

The *Citrus reticulata* peel extract at pH = 5.0 was characterized by using an AVANTES UV-visible spectrophotometer (Apeldoorn, the Netherlands) in the range of 400 to 1000 nm. The powder precursor of *Citrus reticulata* peel and the liquid extract at 3% *w*/*v* were vibrationally characterized by Attenuated total reflectance Fourier transform infrared spectroscopy (ATR-FTIR) using the Lyza 7000 spectrometer from Anton Paar Peru S.A.C. (Lima, Peru), operating from 4000 to 400 cm^−1^ and with a spectral resolution of 2 cm^−1^.

#### 2.4.2. Characterization of the Adsorbent

XRD measurements were carried out using the RIGAKU (Tokyo, Japan) diffractometer under the following optical conditions: λ = 1.54056 Å, 2*θ* from 20° to 80°, a step of 0.02°, 8 s per step, 40 kV, and 30 mA. Using Match v3 software [12], the preliminary phases of the synthesized materials were fully identified. The XRD data were refined using the Rietveld method, and the microstructural information for each sample was obtained. A 200 kV JEOL 2100 FX electron microscope (Tokyo, Japan) was used to obtain morphological and structural properties. High-resolution transmission electron microscopy (HRTEM) images were analyzed using ImageJ 1.54 g software with a minimum count of 600 particles over 10 to 15 images (TEM), from which the particle size distribution (PSD) was obtained. XPS data were acquired using monochromatic Al K_α_ radiation (1486.7 eV, 13 kV, 100 W) and a Specs Phoibos 150 1D-DLD electron analyzer. Zero-field cooling (ZFC) and field cooling (FC) protocols were used to measure the magnetic properties of the samples. The *M*(*H*) loops were recorded at 300 K and 4 K using the VSM module of a Physical Properties Measurement System (PPMS Evercool II from Quantum Design, San Diego, CA, USA). The FC magnetic experiment was conducted using a cooling field of 1 kOe and a sweep field of ±60 kOe. The *M*(*H*) curves were produced at a maximum field strength of 60 kOe for the NPM6 sample. After the dye adsorption experiments, the *M*(*H*) loops of the four selected samples were measured at ±20 kOe. ^57^Fe Mössbauer spectra were collected at 15 K and 300 K in transmission mode using a conventional spectrometer operating in either sinusoidal velocity or constant acceleration with a 25 mCi source of ^57^Co in a Rh matrix. To fit the ^57^Fe spectra, Mosswinn 4.0i [13] or MOSFIT x software was used, involving distributions of quadrupolar doublets and/or magnetic sextets with Lorentzian lines. The velocity of the transducer was calibrated using an α-Fe foil, and the values of isomer shift are referred to that of α-Fe at 300K. The hydrodynamic diameter and zeta potential of the NPM6 sample (used for the dye adsorption experiments) were measured with Brookhaven Nanobrook 90 Plus PALS equipment (NY, U.S.A.) and the particle solution program BIC v.3.6.0.7122 software was used. A NOVA 600 equipment from Anton Paar Peru S.A.C. (Lima, Peru) was used for measurements of the N_2_ adsorption/desorption isotherms at 77 K. Prior to measurements, the degasification process consisted of heating the sample for 3 h at 573 K. The textural properties, including surface specific area (SSA) and pore size distribution, were assessed through a multi-Brunauer–Emmett–Teller (BET) point plot and the density functional theory (DFT) model [14].

### 2.5. Dye Adsorption Experiments

In the adsorption experiments, NPM6 was used with MB and MG dyes. It should be noted that all the mentioned experiments were carried out at 300 K, in triplicate and at a speed of 500 rpm. In the adsorption kinetics tests, 20 mg of the NPM6 sample was applied in 15 mL of MB solution, with an initial concentration of 7 mg L^−1^ at a pH of 5.5. To determine the equilibrium kinetic time, 15 points were used in the range 1 to 60 min. In the case of MG, 25 mg of NPM6 was applied to 15 mL of MG solution with an initial concentration of 8 mg L^−1^ at a pH of 5.5. To determine the equilibrium kinetic time, 17 points were evaluated in the range of 1 to 60 min. Regarding the influence of pH on adsorption, the solution of the two dyes was adjusted to different pH levels (2, 3, 4, 5.5, 6, 7, 8, and 9), applying the NPM6 sample in each of the following conditions: 7 mg L^−1^ MB dye at a dose of 20 mg of NPs in 15 mL of solution for 10 min; and 8 mg L^−1^ MG at a dose of 25 mg of NPs in 15 mL of solution for 25 min.

To determine the adsorption isotherm, a working pH value of 5.5 was chosen at 300 K. For the 7 mg L^−1^ MB dye, the experimental conditions were an equilibrium time of 10 min with a dose of 20 mg NPs in 15 mL of MB dye solution at 15 different initial concentrations ranging from 1 mg L^−1^ to 20 mg L^−1^. For 8 mg L^−1^ MG, an equilibrium time of 25 min was used and a dose of 25 mg NPs in 15 mL of MG dye solution at 11 different initial concentrations, ranging from 1 mg L^−1^ to 15 mg L^−1^.

Adsorbent doses were also evaluated using 5 mg, 10 mg, 15 mg, 20 mg, and 25 mg of NPM6 in 15 mL of 7 mg L^−1^ MB dye solution for 10 min. While 10 mg, 15 mg, 20 mg, 25 mg, and 30 mg of NPM6 in 15 mL of 8 mg L^−1^ MG dye solution for 25 min were tested. The temperature variation in the interaction between adsorbent and adsorbate was monitored at 298 K, 308 K, 318 K, 328 K, and 338 K using a temperature sensor inside the container at a pH of 5.5 and with contact times of 10 min and 25 min for 7 mg L^−1^ MB and 8 mg L^−1^ MG, respectively, and doses of 20 mg and 25 mg in 15 mL of MB and MG dye solution, respectively. Each batch adsorption experiment was conducted with three replicates, and the results were presented as mean ± SD (standard deviation).

The reuse performance of the NPM6 sample was evaluated over seven regeneration cycles, using the following procedure. The sample used in the first cycle was washed with 0.1 M NaOH for 10 min. It was then washed four times, returned to neutral pH, dried at 338 K, and used a second time in the dye solution (MB2 and MG2 samples). This process was repeated until the seventh use (MB7 and MG7 samples). It should be noted that these experiments were carried out in triplicate and according to the optimal parameters established for 7 mg L^−1^ MB at a pH of 5.5 and a dose of 20 mg NPs in 15 mL of dye solution for 10 min and at 300 K. For 8 mg L^−1^ MG, at a pH of 5.5, a dose of 25 mg NPs was added in 15 mL of dye solution for 25 min at 300 K.

To determine and read the concentration for each experiment, a calibration curve was constructed for each dye, either MB or MG. This process involved the preparation of various concentrations of dyes, followed by the measurement of absorbance over a wavelength range of 600 to 700 nm, using the AVANTES UV-visible spectrophotometer (Apeldoorn, the Netherlands). From these data, the respective calibration curves for each dye were derived. At the end of each experiment, a neodymium magnet was placed at the base of the vessel to separate the NPM6, characterized by its magnetic pulling force, to easily treat the aliquot. The resulting sample was transferred to a plastic cuvette and subjected to UV-visible spectrophotometer readings. The absorbance was recorded, enabling the concentration to be calculated.

### 2.6. Adsorption Percentage and Adsorption Capacities for Dyes

For each experiment carried out, the percentage of dye adsorption was calculated as follows:

(1) $$\%R=\frac{C_{i}-C_{t}}{C_{i}}\times 100\%$$

where *C*_i_ (mg L^−1^) is the initial dye concentration and *C*_t_ (mg L^−1^) is the dye concentration at a specific time.

Likewise, the adsorption capacity was calculated experimentally using the following relation:

(2) $$q\left(mgg^{-1}\right)=\frac{\left(C_{i}-C_{t}\right)V}{m}$$

where V (mL) is the volume of the solution and m (mg) represents the amount of adsorbent, respectively.

The theoretical description of non-linear, linear kinetic/isotherm adsorption models, and error analysis is given in the Supplementary Materials, see Section S1.

## 3. Results and Discussion

### 3.1. Citrus reticulata Peel Extract Characterization

**Figure 1:**
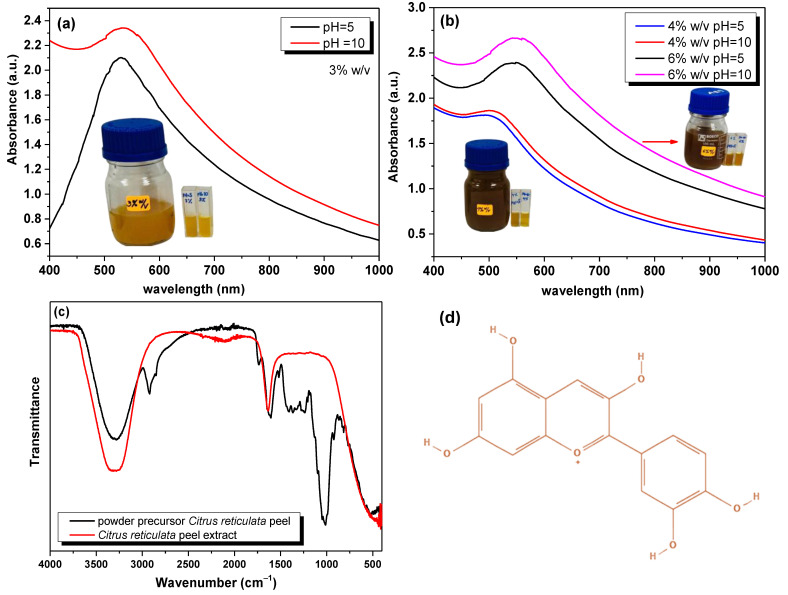
(**a**) UV-Vis spectra of 3% *w*/*v Citrus reticulata* peel extract solution at pH = 5 and 10. (**b**) UV-Vis spectra of 4% *w*/*v* and 6% *w*/*v Citrus reticulata* peel extract solution at pH = 5 and 10. (**c**) FTIR spectra of powder precursor *Citrus reticulata* peel and *Citrus reticulata* peel extract at 3% *w*/*v* (pH = 5). (**d**) Characteristic cyanidin molecule structure at acidic medium. The bottles in (**a**,**b**) indicate the pure *Citrus reticulata* peel extract solutions are at pH = 5, while the small cuvettes at pH of 5 and 10.

The extract’s physicochemical properties were qualitatively studied using UV- Vis spectroscopy. The extract solutions prepared at 3% *w*/*v* (pH = 5) exhibited a pronounced peak at 529 nm, see Figure 1a. This band shifts to 532 nm (Figure 1b) under alkaline medium and has been associated with cyanidin and its derivatives [15]. More precisely, at an acidic pH of 5.5, the structural form of cyanidin [15] corresponds to our results. This is characteristic of the presence of anthocyanins in the liquid extract. However, at high concentrations of 4% *w*/*v* and 6% *w*/*v* (acidic and alkaline medium), the absorption band shifted between 500 and 550 nm. This result is in contrast with the presence of other anthocyanins in the visible region. Despite not having a notorious color change when varying the pH, the results are consistent with the absorbance increasing at high pH values reported in other works [16,17]. The latter is related to concentrated species that absorb more light [16,18]. In this respect, the extract solution at 3% *w*/*v* showed the smallest variation in maximum absorbance. Consequently, it was studied vibrationally by IR spectroscopy in order to elucidate its chemical functional groups. More specifically, the IR active modes of vibration, in Figure 1c, were found [16,19]: (i) the broad and intense IR band at ~3290 cm^−1^ was attributed to -OH stretching vibrations, (ii) the middle intensity IR bands at 2921 and 2851 cm^−1^ were assigned to aliphatic C-H stretching bands, the middle intensity band at 1605 cm^−1^ and the shoulder at 642 cm^−1^ were assigned to aromatic C=C and C-H bonds, C-O groups were also found at 1050 cm^−1^. The peak at 1369 cm^−1^ was attributed to the bending of -OH groups. However, analysis of the extract revealed the absence of the main active chemical groups. This is due to the predominance of the solvent water, as it has also been reported in standard anthocyanins (cyanidin 3-O-glucoside chloride) [16]. Although the -OH (3297 cm^−1^), C=C (1636 cm^−1^), and the C-H (600 cm^−1^) bonds shifted slightly, this indicates that that the dispersive medium affects the vibration properties of cyanidin. As reported in the literature [17,19], the IR spectrum of cyanidin is significantly affected by the drying process or the pH conditions in suspension. The characteristic cyanidin molecule structure at acidic medium is given in Figure 1d.

### 3.2. Effect of Citrus reticulata Peel Extract in the Physicochemical Properties of the Adsorbents

The effect of the concentration of *Citrus reticulata peel extract* was studied to confirm the biosynthesis process using the identified biological substrate (cyanidin). Scheme S1 shows the biosynthesis conditions carried out to obtain six representative samples. Crystallographic phase identification and Rietveld refined parameters are detailed in Figure S1 and Table S1. The NPM1, NPM2, and NPM3 samples, synthesized with mandarin peel extract percentages of 4% *w*/*v*, 6% *w*/*v*, and 3% *w*/*v* (at 300 K), showed Miller indices (100), (012), and (110), characteristic of the 2-line ferrihydrite phase. Regarding their crystallite size, 5 nm, 3 nm, and 3 nm (Table S2) were obtained for the NPM1, NPM2, and NPM3, respectively. Therefore, an increase of the percentage of extract from 4% *w*/*v* to 6% *w*/*v*, results in a reduction in crystallite size. In contrast, the ferrimagnetic (FI) samples (NPM4-6) exhibited featured Bragg’s peaks for inverse spinel structure of iron oxides. The lattice parameters (8.36 Å) indicate that the samples are initially magnetite (Fe_3_O_4_) with mean crystallite sizes of 6 to 10 nm.

It was observed that by increasing the extract concentration to 4 and 6% *w*/*v*, the magnetic behavior results in paramagnetic samples (NPM1-3) with utrasmall sizes of 4 nm, as shown in Figures S2 and S3 and Table S3. The best chemical conditions for synthesis were obtained for in situ (NPs growth in the presence of extract or MWNCTs plus extract) and after NP formation at 3% *w*/*v* and 353 K (NPM4-6 samples) giving FI behavior, as shown in Figure S3. As previously mentioned, the chemical anthocyanin components vary with increasing the extract concentration, which is probably related to the paramagnetic behavior. It is worth noting that the effect of temperature can be also an important factor to be considered for successful biosynthesis, as indicated by the results of the for the NPM3 sample biosynthesized at 300 K and 3% *w*/*v* that showed a paramagnetic behavior. A control sample previously characterized by our group (maghemite control, MC) showed a large size of 15 nm when the 3% *w*/*v Citrus reticulata* peel extract was not used [20]. However, the biosynthesized samples (NPM4-5) had mean particle sizes of 11 and 12 nm. This means that the *Citrus reticulata* peel extract plays an important role in sample dispersity, as shown in Tables S2 and S3. In addition, the magnetization saturation (*M*_s_) values for the in situ samples (NPM4 and NPM6 samples) are close (~55 emu g^−1^), indicating that the presence of MWCNTs did not affect the FI behavior of the magnetic NPs. This value is 17% lower than that reported for the 15 nm maghemite control, related to the anthocyanin content bound to the surface of the NPs.

### 3.3. Preliminary Adsorption Test of the Adsorbents

**Figure 2:**
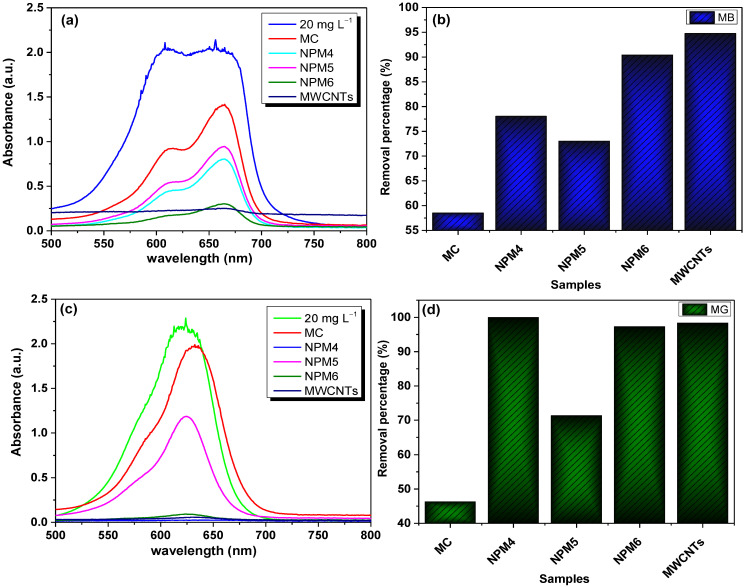
(**a**) UV-Vis adsorption bands for the adsorption experiments performed at 20 mg L^−1^ MB, kinetic time of 10 min, adsorbent dose of 1.3 g L^−1^, 300 K and pH = 6. (**b**) Removal efficiency for MB using the 15 nm maghemite control (MC), NPM4-6, and MWCNTs. (**c**) UV-Vis adsorption bands for the adsorption experiments done at 20 mg L^−1^ MG, kinetic time of 10 min, adsorbent dose of 1.3 g L^−1^, 300 K and pH = 6. (**d**) Removal efficiency for MG using the 15 nm maghemite control (MC), NPM4-6, and MWCNTs. The experiments were performed two times.

**Figure 3:**
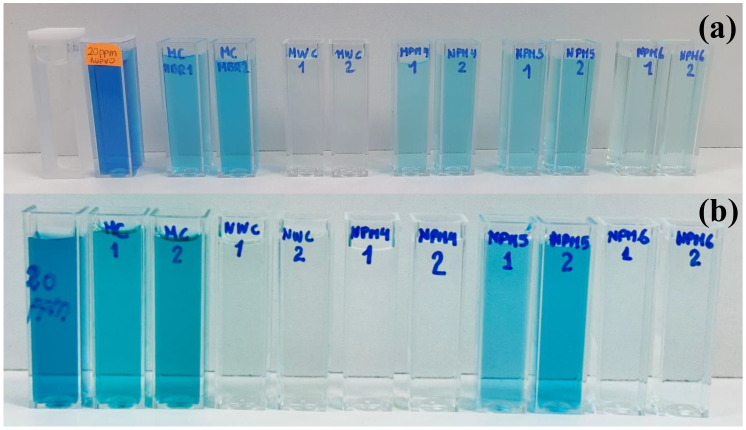
Colorimetric comparison for the preliminary adsorption test for (**a**) 20 mg L^−1^ MB and (**b**) 20 mg L^−1^ MG using the control sample and the biosynthesized adsorbents. 1 and 2 indicate the first and second repetition, respectively.

Before performing different adsorption experiments, the best adsorption performance was evaluated by each FI biosynthesized sample and a maghemite control (MC), as shown in Figure 2a–d. For both dyes, it can be seen that the MC sample has a low removal efficiency, see the absorbance bands and colorimetric comparison in Figure 3. In contrast, the biosynthesized samples showed a higher removal efficiency than the MC control and greater than 70%. MWCNTs showed a removal efficiency equal or greater than 95% for MB and MG. In this sense, the synergistic behavior for dye adsorption is confirmed with the NPM6 sample, which showed removal efficiencies of 90% and 97% for MB and MG. This means that the MWCNTs surface can be magnetically recovered with the biosynthesized NPs without losing their adsorption properties. It is worth mentioning that MWCNTs are complex to handle because they diffused easily into the air, leading to respiratory problems [21]. Consequently, the functionalization with biosynthesized NPs reduces this effect, resulting in a mesoporous magnetic material with remarkable dye adsorption properties. With this purpose in mind, full adsorption studies were done using the NPM6 sample.

### 3.4. Detailed Characterization of the Adsorbent with Best Adsorption Performance

#### 3.4.1. Structural and Morphological Properties of NPM6 Adsorbent

**Figure 4:**
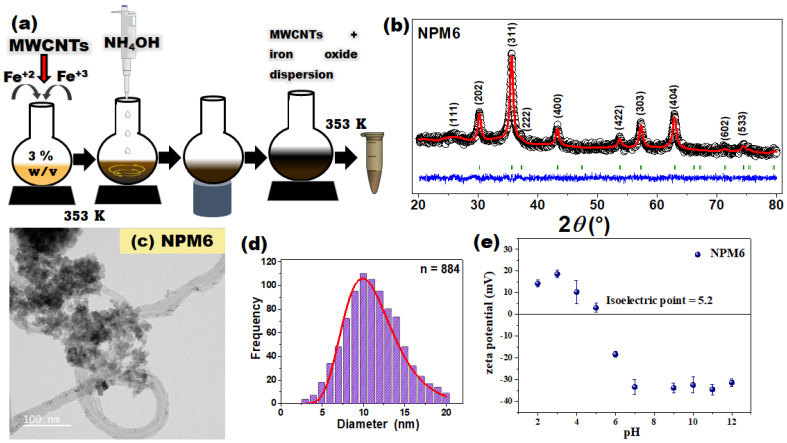
(**a**) Synthesis of iron oxide NPs assisted with *Citrus reticulata* peel extract and functionalization of the MWCNTs. (**b**) Rietveld refinement of the NPM6 sample. In the graphical representation, the black circles correspond to the experimental data, the red lines indicate the calculated diffractogram, the blue lines represent the difference or residual, and the green lines indicate the Bragg reflection positions. Each identified Bragg peak has a Miller index at the top. (**c**) TEM images and PSD histogram of the synthesized NPM6 sample. n in (**d**) indicates the total counted particles to obtain the PSD histogram, and (**e**) titration curve for isoelectric point determination of the NPM6 sample.

Crystallographic phase identification and Rietveld refined parameters are detailed in Tables S1 and S2 and Figure 4b. On the other hand, the NPM6 sample shows a diffractogram with Bragg peaks fully indexed by (202), (311), (222), (400), (422), (303), (602), and (533) Miller indices, a set of peaks found in the γ-Fe_2_O_3_ phase. Their mean crystallite size was calculated at 10 nm. The formation of γ-Fe_2_O_3_ can be explained by assuming that the 3% *w*/*v* mandarin peel extract (used in their syntheses) was sufficient to produce a polyphenol concentration that contributes to the reduction of iron cation species during the biosynthesis process. NPM6 was directly functionalized during synthesis, which may account for the observed larger crystallite size.

The TEM image in Figure 4c shows that the geometry of the NPs is 2D-circular. On the other hand, NPM6, the γ-Fe_2_O_3_@MWCNTs nanohybrid, presented a more defined morphology with cylindrical structures attributed to MWCNTs, and a mean diameter of 11 nm was estimated from the PSD histogram displayed in Figure 4d and Table S3. These results suggest that varying the percentage of extract significantly affects the morphological and dimensional characteristics of the NPs obtained. In Table S3, the polydispersity index of NPM6 sample is reported, a lower polydispersity was also observed, demonstrating the positive influence of NPs on MWCNT surfaces.

#### 3.4.2. Colloidal, Textural, and Surface Properties of NPM6 Adsorbent

The titration curve (zeta potential vs. the pH) was constructed and shown in Figure 4e, and the isoelectric point was regarded at 5.2. The NPM6 sample is stable above this point, reaching values of −33.4 mV at pH = 7. The negative potential is advantageous for increasing the adsorption of the MB and MG cationic dyes. In addition, the isoelectric point is lower than that found in 15 nm MC (7.0), indicating the presence of a different molecular charge around the surface of the NPs, probably related to anthocyanins.

**Figure 5:**
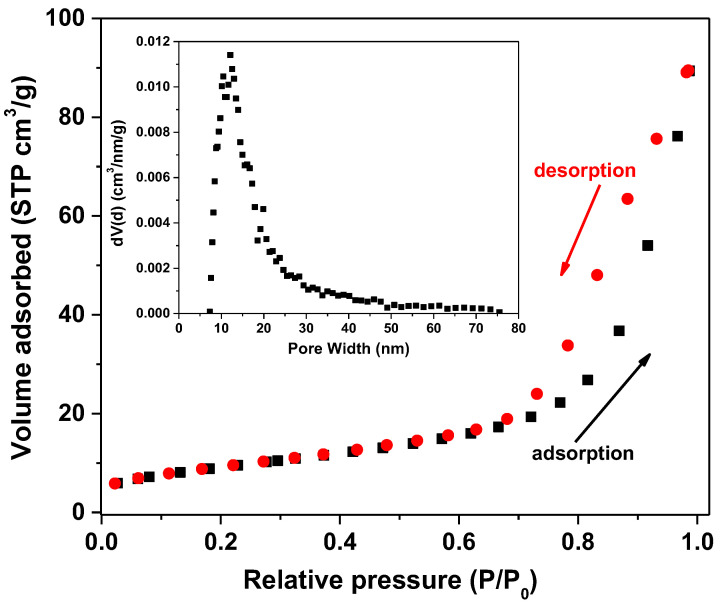
77 K N_2_ adsorption–desorption isotherm for the NPM6 sample. The inset shows the pore width distribution.

The 77 K N_2_ hysteresis adsorption–desorption isotherm depicts an isotherm IV-type, as shown in Figure 5. It is characteristic of mesoporous systems [22]. The pore size distribution obtained from the DFT method shows homogeneous behavior. The physisorption analysis shows a BET surface area of 83 m^2^/g, 0.13 cm^3^/g and pore width of 12 nm. These textural properties supported the results in Section 3.3.

**Figure 6:**
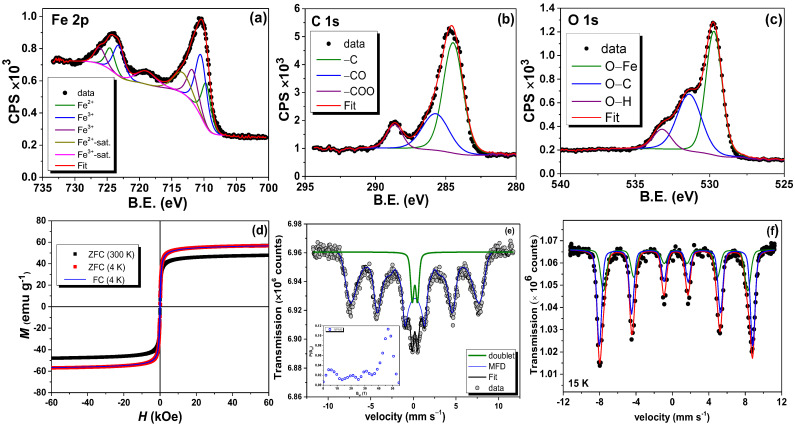
XPS spectra of the Fe 2p, C 1s, and O 1s regions of the NPM6 sample. (**a**–**c**). ZFC and FC *M*(*H*) curves for the NPM6 sample at 300 K and 4 K. *H*_FC_ = 1 kOe. (**d**). (**e**,**f**) ^57^Fe Mössbauer spectra for the NPM6 sample taken at 300 K and 15 K. The black circles represent the data and the red lines the fit. In (**e**) blue line is the MFD and olive line is the doublet (the inset represents the MFD). In (**f**) olive line corresponds to A sites, blue lines to B sites.

XPS measurements are useful to differentiate Fe_3_O_4_ and γ-Fe_2_O_3_ NPs according to the iron valence state [23]. In this case, the biosynthesized magnetic sample showed divalent and trivalent states, indicating the presence of Fe_3_O_4_ NPs in our sample (Tables S4 and S5). For the Fe-2p region, three peaks were used to fit the spectra, located at around 709.7 eV (Fe^2+^), 710.8 eV (Fe^3+^), and 712.2 eV (Fe^3+^). In Figure 6a, the Fe^2+^/Fe^3+^ ratio is 0.53 for the NPM6 sample. These results mean that the NPM6 sample has a higher stoichiometry in the Fe_3_O_4_ phase when using MWCNTs. Despite this, a conventional co-precipitation method (in the absence of extract in the synthesis) only produced Fe^3+^ oxidation states, as previously demonstrated in many systems [24]. Therefore, these results indirectly indicate that mandarin extract has a significant influence on the oxidation process that transforms the Fe_3_O_4_ to the γ-Fe_2_O_3_ phase. In other words, mandarin extract can delay the oxidation process by protecting the NP surfaces for a much longer period.

On the other hand, the C-1s region makes it possible to determine the presence of organic groups linked to polyphenol groups and MWCNTs. In Figure 6b, three functional groups were quantified. The major component for C-C at 284.5–284.4 eV is related to aromatic carbons within phenolic rings or to carbon exoskeleton in MWCNTs. The absence of any characteristic binding energy (B.E.) related to the carbonyl (–C=O) group is highlighted, indicating that no quinone-type compounds are present in the mandarin extract [25]. The O-1s spectrum is shown in Figure 6c and presents three components. Interestingly, the NPM6 sample presents the hydroxyl (OH^−^) group at 532.3 eV, which differs from the expected B.E. position for OH^−^ groups coordinating with benzene rings at 533.6 eV [25]. This shift of 1.2 eV can be interpreted as a possible coordination with Fe ions active on the particle surface. Previous literature concerning the concentration of surface OH^−^ groups on metal oxide films has shown that a hydroxylated region can be formed as an interface between the metal oxide surface and chemisorbed water [26]. The B.E. is expected from 531.9 to 533.9 eV, depending on the coordinating metal (Figure 6c).

When examining the spectra of MWCNTs functionalized with Fe_3_O_4_ NPs, the −COO groups and −CO are around 11 and 28%, respectively. This reduction in minor and intermediate organic groups may be due to the presence and chemical coordination with iron oxide NPs and the predominance of MWCNTs in comparison to phenol groups. In a previous work, the configuration of the monodentate C-O-C bonds was also observed [27]. In our case, this B.E. is located at 531.4 eV for the NPM6 sample (ΔC-O-C = 0.7 eV), hence indicating a surface interaction. It should also be noted that the MWCNTs functionalization process produced a sample of Fe_3_O_4_ NPs with a high stoichiometry. Nevertheless, as shown by the ^57^Fe Mössbauer results and magnetization data discussed, the Fe-oxide phase of the NPM6 sample was oxidized after 4 months of storage at normal laboratory conditions (293 K and 1 atm), independently of the coating and functionalization.

#### 3.4.3. Magnetic and Hyperfine Properties

On the other hand, the *M*(*H*) curves at 4 K and 300 K for the NPM6 sample exhibit an S-like shape, a feature associated with an FI state of the γ-Fe_2_O_3_ phase (see Figure 6d and Table S6). The *M*_s_ value at 300 K was found below those found in the bulk-like γ-Fe_2_O_3_ state (60–80 emu g^−1^) [28], suggesting that the presence of MWCNTs reduces the magnetic response of γ-Fe_2_O_3_ NPs. In addition, the comparison of ZFC and FC *M*(*H*) loops taken at 4 K did not show a large difference and no horizontal loop shift effect that could be attributed to the exchange bias phenomenon in the NPM6 sample (the possible exchange coupling between spins at the surface and core of the particle can be considered negligible).

The squareness ratio (*M*_r_/*M*_s_) was also calculated for both temperatures. The NPM6 sample showed values below 0.5, as expected by Stoner–Wohlfarth theory for an ensemble of magnetic NPs with random anisotropy [29]. More precisely, this result suggests the absence of uniaxial single magnetic domains in the sample. Specifically, this squareness ratio has a characteristic value for interacting particles with a multidomain magnetic structure [30], a result that is indirectly in agreement with that obtained from TEM images that describe agglomerated NPs with a broad PSD.

^57^Fe Mössbauer spectra were carried out for the NPM6 sample at 300 K and 15 K. The spectra, fitted using the model described below, are shown in Figure 6e,f.

The ^57^Fe Mössbauer spectrum at 300 K for NPM6 is composed of a hyperfine field distribution (MFD) related to magnetically blocked large NPs and one quadrupolar doublet related to small NPs [31], see Table S7. It is worth mentioning that depending on the distribution of NPs the fit may give rise to a complex issue due to the presence of superparamagnetic relaxation phenomena, leading to the broadening of the resonant absorption lines. Sometimes the result is only a mathematical representation of the sample, to avoid overestimation of the magnetic components. The mean δ values of the components are equal and far from the values of Fe^2+^ spin states in the NPM6 sample, indicating that the Fe_3_O_4_ phase that could be obtained during co-precipitation oxidized. The ^57^Fe Mössbauer spectra at 15 K of the NPM6 sample also show six relatively broad absorption lines; however, they can now be fitted with two featured static magnetic sextets found in the cubic spinel structure of the γ-Fe_2_O_3_; see the refined magnetic parameters in Table S8. Therefore, given the atomically ordered state suggested by the magnetization data and the two sextets of the spinel structures for the Fe-oxide phase in the sample, it can be inferred that γ-Fe_2_O_3_ NPs can be associated with a FI state in the blocked state [27].

### 3.5. Batch Adsorption Analysis Using NPM6

From the previous characterization, the NPM6 sample exhibited small particle size, low polydispersity, rich anthocyanin phenolic surface, surface enhancement by MWNCTs and notable magnetic strength (tests with a magnet showed easy manipulation of the nanohybrid dispersed in a synthetic water). In addition, its biosynthesis process was characterized using a low percentage of mandarin peel extract (3% *w*/*v*). Hence, to begin with adsorption kinetic experiments, it is worth noting that the major literature reports adsorption batch experiments with initial concentrations that do not exceed 50 mg L^−1^ for MB and MG dyes [32,33,34,35,36,37,38,39,40,41,42,43,44,45,46,47]. Even in natural water, a low concentration of 10 mg L^−1^ has been tested for MB [33]. In this sense, our experiments were done with low dye concentrations, taking into account the problem of environmental pollution and the storage of dye solutions on a laboratory scale.

On the other hand, the Dynamic Light Scattering (DLS) technique (see Figure S4) was used to assess the hydrodynamic particle diameter of the NPM6 sample, i.e., the effective hydrodynamic diameter in aqueous suspension, which was found to be 220 nm. It should be noted that this value includes the real size of the particles and an additional thickness due to the charges on their surface in contact with the water.

#### 3.5.1. MB Removal Performance

Previous research has identified a variety of materials used in MB cleaning, ranging from synthetic and biosynthetic compounds to nanomaterials (see Table 1). For example, the MB adsorption capacity in this research was higher than that reported by Demirezen et al. [32], and the percentage removal was higher than the value found in bulk samples, as shown in Table 1, except for Obayomi et al. [33] and Munagapati et al. [34]. With regard to the contact time of the experiments (i.e., the time the adsorbent was present in the polluted synthetic water), this activity took 10 min, a significantly shorter time than other studies presented in Table 1, with the exception of Bhakta et al. [35], who reported 1 min, and Samaraweera et al. [36], who used a high adsorbent dose of 50 mg.

**Figure 7:**
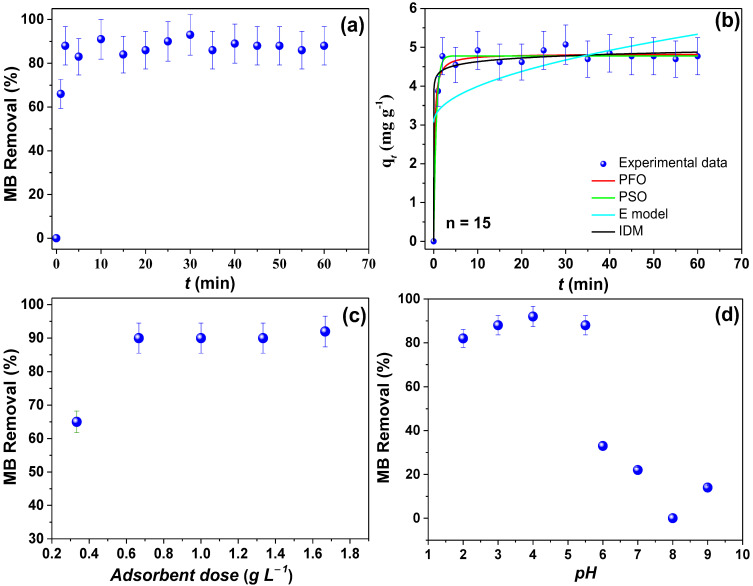
MB removal under different conditions (**a**) MB removal times; (**b**) kinetic fit models; (**c**) effect of adsorbent dose; and (**d**) effect of pH. n here indicates the number of experimental points.

Figure 7a suggests that after 10 min, approximately 90% of the MB dye had been removed from the contaminated synthetic water. The removal efficiency is maintained up to 60 min, a result that can be analyzed with the results reported by Ai and Jiang [43]. In their study, MB removal by a graphene–carbon nanotube hybrid reached its maximum after 120 min of interaction. The adsorption kinetics (Figure 7b) show that after 10 min, the MB dye elimination starts to be constant. The best-fitting kinetic models are Pseudo-First Order (PFO) and Pseudo-Second Order (PSO), with R^2^ values of 0.987 and 0.981, respectively (see Table 2). In addition, thanks to the BIC analysis (which allows us to compare the fits with different models), it can be seen that the PSO has a lower value than other kinetic adsorption models. However, the difference between the two models is not greater than 2. Consequently, both kinetic models can explain that the adsorption mechanism between the adsorbate and adsorbent is chemisorption and that the NPs have a heterogeneous surface (see Table 2). It can be observed that at a higher adsorbent dose of 0.7 g L^−1^ (Figure 7c), elimination is around 90%. The medium with the best elimination is pH 5.5 (Figure 7d).

**Table 2:** Kinetic parameters obtained from fits using kinetic non-linear models for MB adsorption kinetics.

	PFO		PSO		Elovich		IDM	
**Parameters**	*q*_e_	4.78 (0.04)	*q*_e_	4.31 (0.05)	α	6.3 (0.8) × 10^12^	*k*_p_	0.29 (0.11)
	*k*_1_	1.7 (0.2)	*k*_2_	1.1 (0.3)	β	7 (2)	*C*_1_	3.1 (0.6)
**R^2^**	0.987		0.981		0.970		0.297	
**RSS**	23.906		24.550		22.515		24.050	
**BIC**	12.769		12.396		11.011		9.790	

#### 3.5.2. MG Removal Performance

It is worth mentioning that several methods are used to remove synthetic dyes from MG, in particular, the green methyl contaminant, including adsorption, photocatalysis, and electrocatalysis. Table 3 describes some reported works in the literature.

The removal percentage and adsorbent doses are given in Table 3; the values are very close to those reported in this work, and the only significant difference is the shorter time used for removal (25 min).

**Figure 8:**
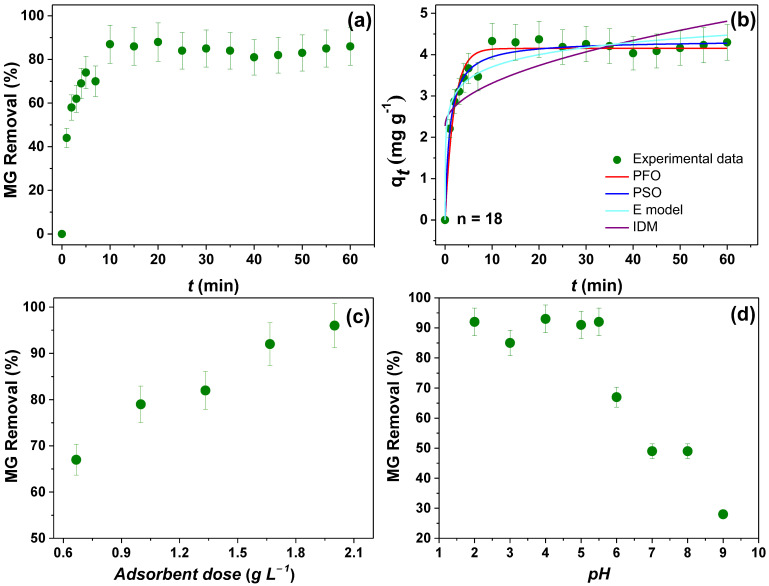
MG removal under different conditions (**a**) MG removal times; (**b**) kinetic fit models; (**c**) effect of adsorbent dose; and (**d**) effect of pH. n here indicates the number of experimental points.

Figure 8a shows that after 10 min, the MG dye elimination percentage exceeds 90%. After 25 min, it remains in the 84–87% region, indicating again a saturation regime. Figure 8b shows that of all the changes in the MG adsorption kinetic model, PFO was the best fit, with R^2^ of 0.95 and a lower BIC than PSO (see Table 4). Furthermore, as the adsorbent dose increases, the removal percentage increases (Figure 8c), with 96% removal at 2 g L^−1^, and the pH at which removal is highest is 5.5 (Figure 8d).

So far, it is worth mentioning that, although the BET surface area of the NPM6 sample (83 m^2^/g) is 25% lower than that of MWCNTs (110 m^2^/g), the biosynthesized magnetic sample achieved remarkable removed efficiency for the dyes tested and competitive adsorption BET area compared with other adsorbents [34,45].

#### 3.5.3. Effect of Temperature and Isotherm Adsorption

**Figure 9:**
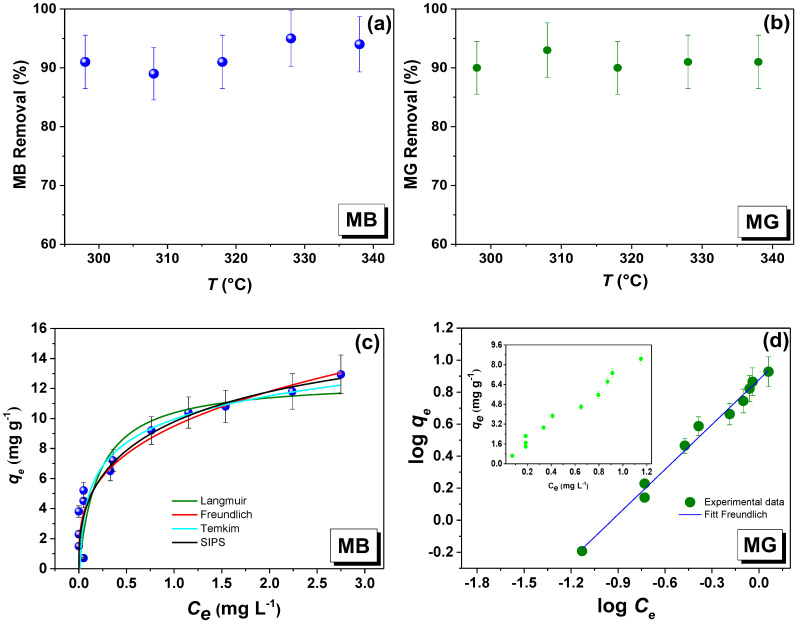
(**a**) Effect of temperature on MB dye; (**b**) Effect of temperature on MG dye; (**c**) MB adsorption isotherm (**d**) adsorption isotherm for MG.

Figure 9a,b displays that the temperature change did not significantly affect the removal percentage of MB and MG. With respect to the adsorption isotherms, for MB, the data were fitted with four non-linear models. The best-fitting models were the Freundlich and Temkim, with R^2^ of 0.81 and 0.80, respectively. Considering the BIC and the two fitted models, the one with the lowest value was the Freundlich model (see Figure 9c), from which the highest adsorption capacity of 9.5 mg g^−1^ is extracted (see Table 5). This model indicates the heterogeneity adsorption that occurs on the surface of the adsorbent [33].

In the case of the MG dye, the behavior of the adsorption isotherm was linear (see Figure 9d), for which linear isotherm models were used (see Table 6). The Langmuir and Freundlich models showed a higher R^2^ of 0.992 and 0.987, respectively, with the Freundlich model having a lower BIC. It showed a maximum adsorption capacity of 7.96 mg g^−1^ (see Figure S5). This model is based on how (i) a single solute adheres to a surface and (ii) the solute splits between the water and the adsorbent surface when they reach equilibrium. Unlike the Langmuir model, the Freundlich model is not limited to the hypothesis of single-layer formation. According to this model, the amount of adsorption can be infinite [48].

#### 3.5.4. Reuse Experiments

**Figure 10:**
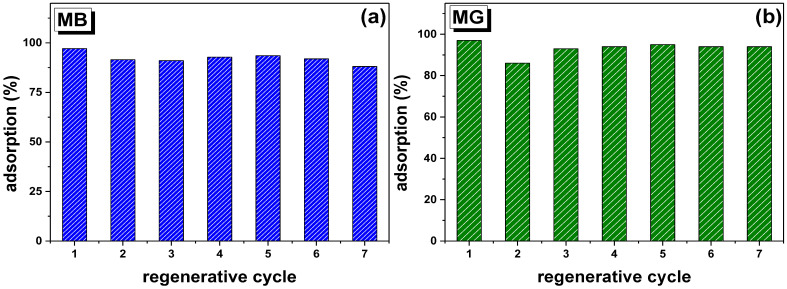
Dyes adsorption with NPM6 in seven regeneration cycles (**a**) MB and (**b**) MG.

During the seven cycles of reuse, the percentage of adsorption was observed to vary between 90% and around 100% for both dyes. In the case of the MB dye, 97% efficiency was achieved on the first use. For the second and third uses, it reduces to 91%. On the fourth and fifth uses, it increased to 92% and 93%, respectively. On the sixth use, it returned to 91%. However, on the seventh use, a significant difference appeared, with a drop to 88%. This suggests that, for the last cycle of use, there may be fewer sites available in NPM6 than in previous cycles (Figure 10a). On the other hand, given the 2% uncertainty (previously established in repeated experiments) on removal efficiency, for all cycles, the nanoadsorbent showed remarkable removal properties, favoring its application to remediation, since an adsorbent must have a property to be reused efficiently, like the present material.

For reuse in MG removal, a reduction in adsorption efficiency is evident between the first and second use, at 97% and 86%, respectively. On the other hand, the third use increased again to 93%, probably due to the adequate washing with 0.1 M NaOH. In other words, this reagent removed the MG from the surface of the nanomaterial, allowing further efficient removal. From the fourth use onward, the percentage removal was between 94 and 95% (see Figure 10b). Once again, the NPM6 magnetic adsorbent showed good efficiency in its reuse for MG removal. Consequently, it has been shown that it can be used for magnetic remediation of MG and MB dye contaminations.

Table 7 shows a comparison with other systems, showing that ethanol and NaOH have generally been used to wash the materials to be reused, mainly associated with their low prices in comparison to that of expensive organic materials, easy to prepare and handle characteristics in laboratory conditions, which supports the present study using 0.1 M NaOH. In the reported works, four cycles are generally used; however, it would be desirable to carry out more than seven cycles to find out to what extent the material continues to act as an adsorbent and even whether its magnetic, physical, chemical, and morphological properties change. Therefore, given the above tests and an efficiency of around 93%, the NPM6 sample looks promising.

### 3.6. Physicochemical Properties of NPs After Adsorption

The characterization of the material after it has been applied to remove the dyes is important to be able to propose the interaction mechanism between the adsorbent and the adsorbate in the dyes removal, and for this XPS, VSM, and ^57^Fe Mössbauer experiments were carried out.

#### 3.6.1. XPS After Adsorption Analysis

**Figure 11:**
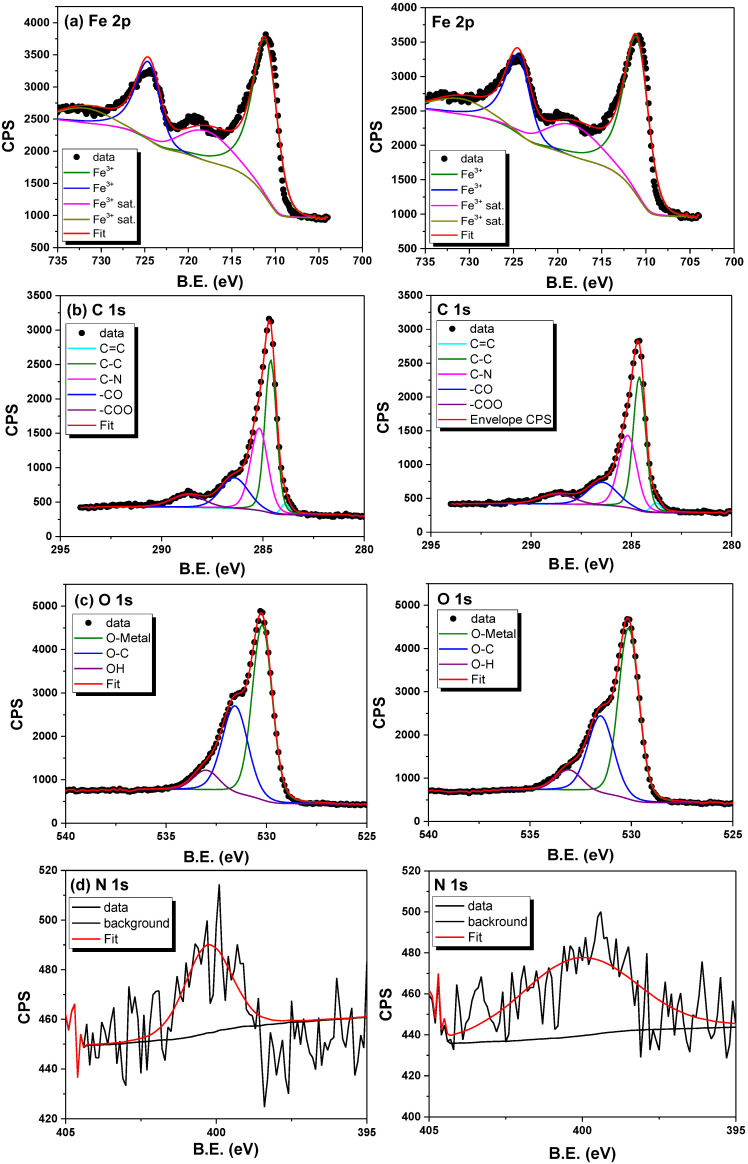
X-ray photoelectron spectroscopy (XPS) analysis, contributions of (**a**) Fe2p, (**b**) C1s, (**c**) O1s, and (**d**) N1s. The left column corresponds to the material recovered from the second use with the MB dye (MB2), and the right column corresponds to the seventh use with the MB dye (MB7).

**Figure 12:**
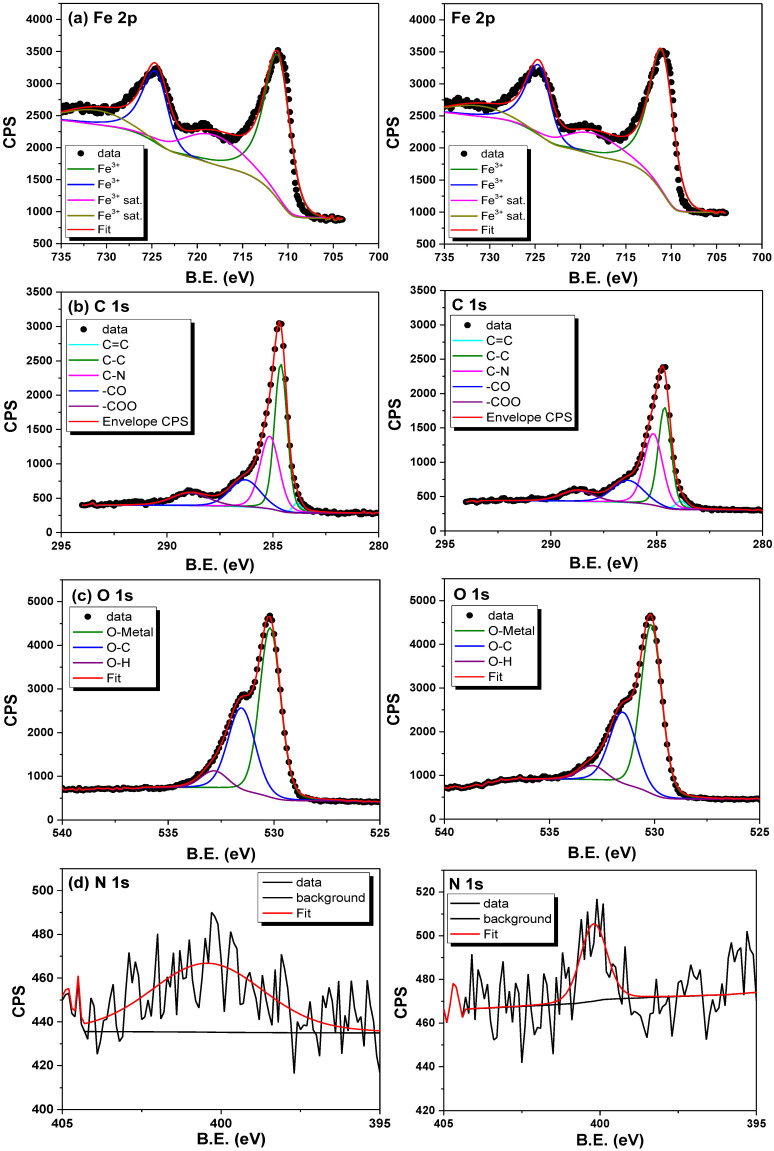
X-ray photoelectron spectroscopy (XPS) analysis, contributions of (**a**) Fe2p, (**b**) C1s, (**c**) O1s, and (**d**) N1s. The left column corresponds to the material recovered from the second use with the MG dye (MG2), and the right column corresponds to the seventh use with the MG dye (MG7).

The post-adsorption behavior of MB and MG recovered from the second and seventh regeneration cycles was studied by XPS. First, it must be pointed out that the iron valence state has changed during exposure to air, as also shown by ^57^Fe Mössbauer spectrometry, i.e., the Fe_3_O_4_ NPs in the NPM6 sample were oxidized. As a result, the spectra of all four samples show a strong peak at 710.9 eV, a main feature of Fe^3+^ states. More importantly, Figure 11 and Figure 12 for MB2(MG2) and MB7(MG7) samples show the same spectra, confirming the efficiency of the reuse properties found for the NPM6 sample during the second and seventh regeneration cycles. In addition, the chemical formula of MB (C_16_H_18_N_3_SCl) is similar to that of MG (C_26_H_33_Cl_2_N_3_) [49,50], where only the characteristic B.E. peaks for C-1s and N-1s regions were observed. In contrast to the initial NPM6 sample, two additional components at 283.9 eV (C=C) and 285.2 eV (C-N) were observed. The C=C component may originate from MB(MG), which has aromatic rings in the structure and contains sp^2^ carbon [47]. The B.E. value of 400.3 eV implies the existence of a p-conjugated N-heterocyclic system positioned at the center of the molecular structure [49]. During the adsorption process, electrostatic forces, π–π stacking, and chemical bonding can have an impact on the overall stability of the MB(MG) molecule, and consequently, its sulfur and chlorine contents. In our case, the absence of the S and Cl atoms suggests that the molecular structure of MB(MG) has undergone partial alteration during the adsorption process. Therefore, it can be assumed that the non-participation of these atoms occurs during the adsorption process [51] (see Table S9).

#### 3.6.2. VSM After Adsorption Analysis

Magnetometry analysis of the vibrating sample of the material recovered in the second and seventh uses of the two dyes, MB and MG, shows the *M*(*H*) curves characteristic of FI materials [52]; see Figures S6 and S7.

The material recovered from the second use (after the interaction with MB) shows a magnetization at 5 K of 23 emu g^−1^, and after the seventh use, this has increased to 40 emu g^−1^ (see Table 8), indicating a relative increase in the magnetic response of the sample. Conversely, after the second use in the MG removal process, magnetization was 38 emu g^−1^, and after the seventh use, it fell to 4 emu g^−1^. Thus, it can be deduced that the consecutive regeneration processes of the NPM6 sample after MB and MG removal were successful done and the *M*_s_ values are reduced due to MB and MG adsorption with respect to the NPM6 sample (~46 emu g^−1^ at 300 K); however, the removal percentages remained around 93% on average, and the sample’s magnetism is still important to be attracted by commercial permanent magnets.

#### 3.6.3. ^57^Fe Mössbauer Analysis

**Figure 13:**
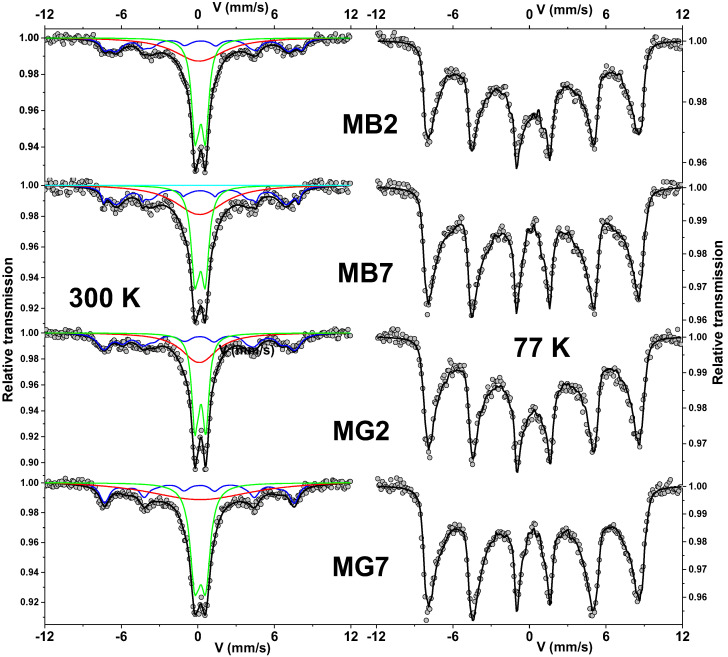
^57^Fe Mössbauer spectra for MB2, MB7, MG2 and MG7 samples obtained at 300 and 77 K. The black circles represent the data and the black lines the fit. Red, blue and green lines are explained in the text.

The Mössbauer spectra obtained at 300 and 77 K for MB2, MB7, MG2 and MG7 samples are shown in Figure 13. At 300 K, each of the hyperfine structures consists of a central quadrupolar component and a magnetic component with broad lines. They can be described using three main components: a quadrupolar doublet, a single broad line and a magnetic component with broad lines. They can be attributed, respectively, (i) to ultra-small, isolated NPs exhibiting very rapid relaxation phenomena, (ii) to weakly larger interacting NPs and (iii) to larger NPs, some of them showing a blocked magnetic structure. At 77 K, the hyperfine structures are rather similar, showing a magnetic component with broad and asymmetrical lines. They can be described using a distribution of hyperfine fields linearly correlated with that of isomer shift to take into account the weak asymmetry in the intensity of the outer peaks. These results corroborate those obtained at 300 K, giving evidence of a progressive blocking of the magnetic structures of the NPs and a distribution of their sizes. In addition, it is important to note that the evolution of the central part of the Mössbauer spectra is consistent with an increase in the size of the NPs after the seventh regeneration cycle.

### 3.7. Surface Adsorption Mechanism

The adsorption of MB onto MWCNTs and magnetic NPs involves complex processes influenced by a diverse range of physical and chemical interactions [53,54]. Firstly, zeta potential measurements at different pH values for the NPM 6 sample revealed a p.z.c. equal to 5.2, as shown in Figure 4e, and the optimum pH value for adsorption experiments was 5.5, indicating a predominance of negative charges interacting with MB chemical groups. At the same time, chemisorption is facilitated by specific interactions between the functional groups on MWCNTs (carboxyl and hydroxyl) and the equivalent cationic groups on the MB(MG) dye. In a previous research, electrostatic adsorption behavior has been postulated for magnetite-loaded multi-wall carbon nanotubes [54]. In addition, MWCNTs involve pore filling within the nanostructure and π–π stacking interactions with the aromatic benzene rings of the dye [54].

**Scheme 2:**
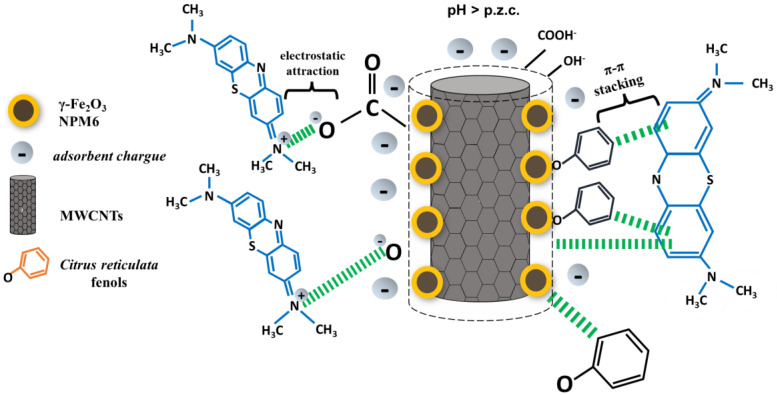
Pictorial representation for the MB(MG) adsorption mechanism onto NPM6 sample.

The aromatic behavior of the phenolic groups on the γ-Fe_2_O_3_ NPs also favors π–π stacking interactions with the aromatic rings of MB(MG), while the cationic nature of MB(MG) favors electrostatic attraction with the negative surface charge of the NPs (hydroxyl groups). Therefore, the adsorption mechanism of MB(MG) involves the following steps: (i) electrostatic adsorption due to the negative surface groups of the adsorbent and the cationic groups, and (ii) π–π stacking interactions with the benzene aromatic rings of the dye, the hexagonal skeleton of the MWCNTs and the phenolic ring groups of the biosynthesized sample. Scheme 2 shows the MB(MG) adsorption mechanism using the NPM6 sample.

## 4. Conclusions

*Citrus reticulata* peel extract, enriched in polyphenols (cyanidin determined from UV-Vis absorbance band at 529 nm), plays a key role in reducing the biosynthesis of 11 nm iron oxide NPs. The effect of the extract concentration clearly had an impact on the magnetic properties of the obtained samples (4 nm ferrihydrite and 11 nm maghemite). Functionalizing these FI maghemite NPs on MWCNTs, not only increased the efficiency of MB/MG removal, but also allowed the loading of anthocyanin chemical groups and the formation of magnetic complexes with dye molecules. In adsorption tests, 93% MB and 84% MG were removed in 10 and 25 min, respectively. The NPs were successfully recovered and could be reused in 7 cycles, maintaining a high adsorption percentage (around 90% for MB and MG). The dye adsorption process can be described as a two-step mechanism. First, there is electrostatic adsorption, where the negative surface groups from the adsorbent (with an isoelectric point of 5.2) interact with the cationic groups of the dye. Second, there are π–π stacking interactions between the aromatic benzene rings of the dyes, the hexagonal skeleton of the MWCNTS and the phenolic ring groups of the biosynthesized sample. Thus, the improved magnetic nanoadsorbent shows remarkable and competitive adsorption properties for polluting dyes and can be used for the alternative bioremediation treatment of polluted effluents.

## Figures and Tables

**Table 1:** Main adsorption parameters for parent adsorbent materials used in MB removal. ND: not defined.

Sample	Adsorption Capacity (mg g^−1^)	%	pH	Time (min)	Dose	Reference
SDS-CSMAB Chitosan/Calcium Alginate Double Network Magnetic Hydrogel Beads	1.9	61	5.5	60	0.1 g L^−1^	[32]
CSAC@AgNPs@TiO_2_ NP	184	97	10	60	30 mg	[33]
Fe_3_O_4_-GLP@CAB Magnetic Fe_3_O_4_ NPs loaded guava leaves powder impregnated into calcium alginate hydrogel beads	137	95	10	90	1 g	[34]
MWCNTs + γ-Fe_2_O_3_ NPs	59.4	-	ND	1	0.3 g L^−1^	[35]
Fe_3_O_4_-modified Douglas fir biochar (FDBC)	741	90	6.8	5	50 mg	[36]
CdS/SnO_2_ heterostructured NP	-	90	photocatalytic degradation	180	0.1 g	[37]
Biochar/FexOy composite	862	-	6.1	720	0.01 g	[38]
NP CN-Fe_2_O_3_ Green synthesis	530.4	88	5	60	25 mg L^−1^	[39]
IONPs Green synthesis	-	80	ND	30	2 mg	[40]
Biochar-Fe_x_O_y_ date palm frond	85.1	-	8	198	0.02 g	[41]
Fe_3_O_4_ *P. factra* extract	26.8	91	9	50	0.7 g L^−1^	[42]
NPM6	9.5	93	5.5	10	1.3 g L^−1^	This work

**Table 3:** Reported adsorbent materials used in MG adsorption.

Sample	Adsorption Capacity (mg g^−1^)	%	pH	Time (min)	Dose	Reference
BC	5.6	95	2–9	50	0.9 g	[44]
*Tamarindus indica* fruit seed nanomagnetic/biocarbon composite (nM-BC)	20.4	99	2–9	50	0.9 g	[44]
adsorbent chitosan/Fe_2_O_3_/NiFe_2_O_4_	77.2	97	8	120	0.02 g	[45]
graphene oxide (CuSe/GO)	-	81	5	45	0.025 g	[46]
Fixed bed adsorption MCM-41	21	50	6	-	0.8 mL min^−1^	[47]
NPM6	8	84	5.5	25	1.7 g L^−1^	This work

**Table 4:** Kinetics parameters obtained from fits using kinetic non-linear models for MG adsorption kinetics.

	PFO		PSO		Elovich		IDM	
**Parameters**	*q*_e_	4.15 (0.07)	*q*_e_	4.35 (0.06)	α	244.2 (2)	*k*_p_	0.33 (0.08)
	*k*_1_	0.54 (0.05)	*k*_2_	0.23 (0.03)	β	2.3 (0.3)	*C*_1_	2.3 (0.4)
**R^2^**	0.95		0.98		0.93		0.51	
**RSS**	25.07		29.52		24.08		21.24	
**BIC**	11.74		14.69		11.02		5.87	

**Table 5:** Isotherm parameters obtained from fits of the data of the NPM6 sample using nonlinear adsorption isotherm models for the MB.

Langmuir		Freundlich		Temkim		Sips	
***q*** **_max_**	13 (1)	*K*_F_	9.5 (0.6)	*b*_T_	2.25 (0.37)	*q*_ms_	2 (1) × 10
***K*** **_L_**	5 (2)	*n*	0.32 (0.06)	*K*_T_	8 (5) × 10	*K*_s_	0.3 (0.9)
						*m_s_*	0.4 (0.2)
**R^2^**	0.775	R^2^	0.81	R^2^	0.80	R^2^	0.795
**BIC**	46.041	BIC	56.049	BIC	118.04	BIC	55.79

**Table 6:** Isotherm parameters obtained from fits of the data of the NPM6 sample using the linear adsorption isotherm models for the MG.

Langmuir		Freundlich		Temkim		Sips	
*b*	0.17 (0.02)	1/n	0.88 (0.03)	*K*_T_	2.9 (0.4)	1/n	0.8 (0.2)
*q*_max_	8.72 (0.01)	*K*_F_	7.96 (0.05)	*b*_T_	10.8 (0.3)	*b*_s_	7.9 (0.2)
R^2^	0.992	R^2^	0.987	R^2^	0.888	R^2^	0.926
BIC	36.19	BIC	34.46	BIC	43.41	BIC	34.21

**Table 7:** Other NP reuse systems in MB and MG adsorption.

Systems	Number of Reuses	%	Desorbing Reagent	Reference
SDS-CSMAB/MB	2	61	surfactant loaded CSMAB.	[32]
CSAC@AgNPs@TiO_2_NPs/MB	6	99.33–86.97	ethanol mixed with acetic acid, and ultrapure water	[33]
Biochar-Fe_x_O_y_/MB	4	85 to 39.1 mg g^−1^	ethanol and filtered	[41]
nM- BC/MB	4	88.36 to 86.74	0.1 M NaOH	[44]
nM-BC/MG	4	91.25 to 88.43	0.1 M NaOH	[44]
chitosan/Fe_2_O_3_/NiFe_2_O4/MG	5	97 to 91	-	[45]
MCM-41 fixed-bed regeneration/MG	4	44.38 to 32	0.1 M NaOH	[47]
NPM6/MB	7	93 to 88	0.1 M NaOH	This work
NPM6/MG	7	97 to 94	0.1 M NaOH	This work

**Table 8:** Magnetic parameters of the NPM6 sample after its application in MB and MG dyes.

Sample	T (K)	*M*_r_ (emu g^−1^) ± 0.1	*H*_c_ (Oe) ± 3	*M*_s_ (emu g^−1^) ± 1	*M*_r_/*M*_s_	χ 10^−4^ (emu/g kOe) ± 0.1	*K*_eff_ × 10^5^ (J m^−3^) ± 0.1
MB2	5	9.0	300	23	0.39	1.3	0.6
	300	1.4	40	16	0.09	1.5	0.5
MB7	5	16.1	340	40	0.41	2.4	1.2
	300	1.6	20	29	0.05	2.4	0.9
MG2	5	14.5	310	38	0.38	2.1	1.1
	300	2.3	40	27	0.08	2.2	0.8
MG7	5	1.7	340	4	0.40	0.3	0.1
	300	0.2	30	3	0.05	0.3	0.1

## Data Availability

The original data related to this research can be requested at any time.by sending an email to the corresponding author: juan.ramos5@unmsm.edu.pe.

## Supplementary Materials

The following supporting information can be downloaded at: https://www.mdpi.com/article/10.3390/nano15080603/s1. Theoretical background of non-linear kinetic and isotherm adsorption models. Table S1. Rietveld refined parameters of structural properties of the NPM1-6 samples. Table S2. Crystallite size and Caglioti parameters. Table S3. Statistical parameters for NPM1-6 samples obtained from the PSD histogram. Dm: diameter of particle, SD: standard deviation, PDI: polydispersity index. Table S4. Magnetic parameters were obtained for the NPM1-6 samples. *M*_r_ is the remanent magnetization, *H*_c_ is the coercive field, χ is the paramagnetic susceptibility, and *K*_eff_ is the effective anisotropy constant. Table S5. Elemental Characterization by XPS: Bond Energy Profiles and Surface Composition of NPM6. Table S6. Elemental composition of the elements found in the NPM6 sample by XPS. Table S7. Hyperfine parameters for the NPM6 sample. RAA: relative spectral absorption area, δ: isomer shift vs. Fe at 300 K; *B*_hf_: Magnetic hyperfine field; QS: Quadrupole splitting (fixed); σ: width of Gaussian distribution of *B*_hf_; W: Lorentzian width (fixed = 0.24 mm s^−1^). Table S8. Hyperfine parameters for the NPM6 sample. RAA: relative spectral absorption area, δ: isomer shift vs. Fe at 15 K; *B*_hf_: Magnetic hyperfine field; Q: Quadrupole splitting (fixed); σ: width of Gaussian distribution of *B*_hf_; W: Lorentzian width (fixed = 0.24 mm s^−1^). Table S9. Elemental composition of the elements found in the after-adsorption samples by XPS. Scheme S1. (**a**) Synthesis of iron oxide NPs assisted with *Citrus reticulata* peel extract. (**b**) NPM1-6 synthesized samples and classified into paramagnetic: NPM1, NPM2, and NPM3; and (**c**) Ferrimagnetic: NPM4, NPM5, and NPM6. Figure S1. Rietveld refinement of the NPM1-5 samples. In the graphical representation, the black circles correspond to the experimental data, the red lines indicate the calculated diffractogram, the blue lines represent the difference or residual, and the green lines indicate the Bragg reflection positions. Figure S2. TEM images and size distribution of the synthesized NPM1-5 samples. n indicates the total counted particles to obtain the PSD histogram. Figure S3. ZFC and FC *M*(*H*) curves for NPM1-5 samples at 300 K and 4 K. *H*_FC_ = 1 kOe. (**a**–**c**) paramagnetic and (**d**,**e**) ferrimagnetic samples. Figure S4. Lognormal distribution of hydrodynamic diameter of NPM6. Figure S5. Linear fits of adsorption isotherm for the MG dye. Figure S6. *M*(*H*) curves of NPM6 recovered in the second (2nd) and seventh (7th) use cycles of MB (**a**) at 300 K after the 2nd use, (**b**) at 5 K after the 2nd use, (**c**) at 300 K after the 7th use, and (**d**) at 5 K after the 7th use. Figure S7. *M*(*H*) curves of NPM6 recovered in the second (2nd) and seventh (7th) use cycles of MG (**a**) at 300 K after the 2nd use, (**b**) at 5 K after the 2nd use, (**c**) at 300 K after the 7th use, and (**d**) at 5 K after the 7th use.
