# Supplementary material for: Fast Kinetic Response and Efficient Removal of Methyl Blue and Methyl Green Dyes by Functionalized Multiwall Carbon Nanotubes Powered with Iron Oxide Nanoparticles and Citrus reticulata Peel Extract
Source: Nanomaterials (Basel). 2025 Apr 14;15(8):603. doi: 10.3390/nano15080603 (PMC12029408; doi:10.3390/nano15080603)
Supplement: Supplementary file 1 [file nanomaterials-15-00603-s001.zip › nanomaterials-3541044-supplementary.pdf]

Supporting information for

Fast Kinetic Response and Efficient Removal of Methyl Blue and Methyl Green Dyes by Functionalized Multiwall Carbon Nanotubes Powered with Iron Oxide Nanoparticles and *Citrus reticulata* Peel Extract

Erich V. Manrique-Castillo [1], Mercedes del Pilar Marcos-Carrillo [1],

Noemi-Raquel Checca-Huaman [2], Bruno L. D. Santos [3], Waldemar A. A. Macedo [3],

César A. Barrero Meneses [4], Edson C. Passamani [5], Jean-Marc Greneche [6]

and Juan A. Ramos-Guivar [1,*]

Corresponding Author: juan.ramos5@unmsm.edu.pe

[1] Grupo de Investigación de Nanotecnología Aplicada para Biorremediación Ambiental, Energía, Biomedicina y Agricultura (NANOTECH), Facultad de Ciencias Físicas, Universidad Nacional Mayor de San Marcos, Lima 15081, Peru; emanriquec@unmsm.edu.pe (E.V.M.-C.); 10130030@unmsm.edu.pe (M.d.P.M.-C.)

[2] Centro Brasileiro de Pesquisas Físicas (CBPF), Rio de Janeiro 22290-180, Brazil; nomifsc@cbpf.br

[3] Centro de Desenvolvimento da Tecnologia Nuclear–CDTN, Belo Horizonte 31270-901, Brazil; bruno.santos@cdtn.br (B.L.D.S.); wmacedo@cdtn.br (W.A.A.M.)

[4] Solid State Research Group, Faculty of Exact and Natural Sciences, University of Antioquia-UdeA, Medellín 050010, Colombia; cesar.barrero@udea.edu.co

[5] Departamento de Física, Universidade Federal do Espírito Santo, Vitória 29075-910, Brazil; edson.caetano@ufes.br

[6] Institut des Molécules et Matériaux du Mans (IMMM UMR CNRS 6283), Le Mans Université, 72085 Le Mans Cedex 9, France; jean-marc.greneche@univ-lemans.fr

Supplementary Sections

S1. Theoretical background of non-linear kinetic and isotherm adsorption models

1. Kinetic adsorption models

1.1. Pseudo-First Order (PFO) kinetic model. This model describes adsorption as a physical process that occurs on the surface and is mathematically represented in Equation S1:

$$q_t = q_e\left(1 - e^{-k_1 t}\right) \quad (S1)$$

Where q_e corresponds to the amount of dye adsorbed per gram of compound at equilibrium (mg g^{-1}), t (min), and k_1 is the PFO rate constant (min^{-1}).

1.2. Pseudo-Second Order (PSO) kinetic model. The adsorption is of the chemisorption type and is described with Equation S2:

$$q_t = \frac{k_2\, q_e^2 t}{1 + k_2\, q_e^2 t} \quad (S2)$$

Where k_2 is the PSO adsorption rate constant (g mg^{-1} min^{-1}).

1.3. Elovich kinetic model. This model postulates that the adsorption rate decreases exponentially as the number of dye molecules increases, following a chemisorption process, described in Equation S3.

$$\frac{dq_t}{dt} = \alpha e^{\beta q_t} \quad (S3)$$

where β (g mg^{-1}) represents the desorption constant related to the surface coverage, while α indicates the initial adsorption rate (mg g^{-1} h^{-1}).

1.4. Intraparticle diffusion model (IDM). This model is based on the transport of adsorbate through the internal pore structure of the adsorbent and subsequent diffusion within the adsorbate, creating a uniformly porous structure within the adsorbent. Equation represents the internal diffusion model (IDM) (See equation S4).

$$q_t = k_p\sqrt{t} + C_1 \quad (S4)$$

where k_p (mg g^{-1} h$^{-1/2}$) is the intraparticle diffusion rate constant, and C_1 represents the thickness of the boundary layer (mg g^{-1}). Specific adsorption varies as a function of the square root of the contact time, and the slope reflects the intraparticle diffusion rate.

2. Adsorption isotherm

2.1. Models nonlinear

2.1.1. The Langmuir isotherm model requires absence of interaction between the adsorbed species, i.e., it indicates that only one species of solute will occupy an active site on the homogeneous surface of the adsorbent. This model is represented by Equation S5:

$$q_{\mathrm{e}} = \frac{K_{\mathrm{L}}\, C_{\mathrm{e}}\, q_{\mathrm{max}}}{1+K_{\mathrm{L}}C_{\mathrm{e}}} \quad \text{(S5)}$$

In the equation, C_e (mg L^{-1}) represents the equilibrium concentration of the adsorbate, q_{max} (mg g^{-1}) indicates the maximum adsorption capacity, and K_L (L mg^{-1}) is the constant associated with the adsorption energy.

2.1.2. Freundlich model does not limit the formation of monolayers or multilayers and postulates that the adsorption process takes place on heterogeneous surfaces. It is formulated by Equation S6:

$$q_{\mathrm{e}} = K_{\mathrm{F}}\, C_{\mathrm{e}}^{\frac{1}{n}} \quad \text{(S6)}$$

In this equation, K_F (mg g^{-1}) (mg L^{-1})$^{-1/n}$ is a constant denoting the adsorption capacity, while n represents the adsorption intensity.

2.1.3. The Temkin model indicates that as the sites on the adsorbent surface increase and a homogeneous distribution of binding energies is achieved until a maximum binding energy is reached, adsorption decreases. It is represented by Equation S7:

$$q_{\mathrm{e}} = \frac{\mathrm{RT}}{b_{\mathrm{T}}} \ln(K_{\mathrm{T}}C_{\mathrm{e}}) \quad \text{(S7)}$$

where R (8.31 J mol^{-1} K^{-1}) is the gas constant, T (K) is the absolute temperature, b_T (J mol^{-1}) is the variation of the adsorption energy, and K_T (L g^{-1}) is the constant equilibrium bond.

2.1.4. SIPS model is a three-parameter model that predicts non-uniform surfaces in adsorption. It is a combination of the Langmuir and Freundlich models. It is described by Equation S8:

$$q_e = \frac{q_{ms} k_s C_e^{ms}}{1 + k_s C_e^{ms}} \qquad \text{(S8)}$$

where q_{ms} (mg g^{-1}) represents the maximum adsorption capacity according to the Sips model, k_s (L mg^{-1}), ms is the equilibrium constant of the Sips model.

2.2.Linear Isotherm Models

For the MG dye, the adsorption isotherms were fitted with the linear forms of the Langmuir isotherm (Equation S9):

$$\frac{C_e}{q_e} = \frac{1}{(b \times q_{max})} + \frac{C_e}{q_{max}} \qquad \text{(S9)}$$

Where q_{max} (mg g^{-1}) is the amount of solutes adsorbed on the surface and b is the adsorption energy.

The isotherm of the Freundlich model was fitted with the Equation (S10):

$$\log q_e = \log K_F + \frac{1}{n} \log C_e \qquad \text{(S10)}$$

Where K_F is the Freundlich constant, and $1/n$ is the adsorption intensity:

The linear Temkim model is given by the Equation (S11):

$$q_e = \frac{RT}{q_e} \ln A_T + \frac{RT}{b_T} \ln C_e \qquad \text{(S11)}$$

Where K_T (L g^{-1}) is the constant equilibrium bond and b_T (J mol^{-1}) is the variation of the adsorption energy:

And the SIPS model was defined by Equation (S12):

$$\log \frac{q_e}{(q_m - q_e)} = \frac{1}{n} C_e + \ln(b_s)^{\frac{1}{n}} \qquad \text{(S12)}$$

Where b_s and $1/n$ are the SIPS constants.

3. Statistical methods in model selection

3.1. Residual Sum Squares (RSS)

The RSS estimates the difference between the predicted and actual values of the dependent variable. The goal is to close the difference; therefore, a lower RSS indicates a better fit between the model and the data. Equation (S13) shows the RSS formula:

$$\mathrm{RSS} = \sum(\mathrm{y_i} - \hat{\mathrm{y}}_\mathrm{i})^2 \qquad \text{(S13)}$$

Where $\mathrm{y_I}$ and $\hat{\mathrm{y}}_\mathrm{i}$ are the actual and predicted values of the dependent variable:

3.2. Bayesian Information Criterion (BIC)

The BIC was used to determine the best kinetic and isothermal models that describe the adsorption data of our material: It is expressed mathematically by Equation (S14):

$$\mathrm{BIC} = n \ln\left(\frac{\mathrm{RSS}}{\mathrm{n}}\right) + \mathrm{k}\ln\,(n) \qquad \text{(S14)}$$

Where *n* are experimental points and *k* is the number of parameters in the model.

Supplementary Tables

Table S1

Rietveld refined Parameters of structural properties of the NPM1-6 samples.

sample	Atoms	x	y	z	Thermal parameters (Occ.)	Lattice parameters (Å)	Statical parameters (%)
**NPM1**	Fe	0.1688	0.8312	0.6356	1.0	a = b = 6.07 c = 9.13	R_P = 62.8 R_{wp} = 43.6 R_{exp} = 40.9 χ^2 = 1.13
	Fe	0.3333	0.6667	0.3414	1.0		
	Fe	0.3333	0.6667	0.9538	1.0		
	O	0.0000	0.0000	0.0147	1.0	α = β = 90° γ=120°	
	O	0.3333	0.6667	0.7353	1.0		
	O	0.1671	0.8329	0.2547	1.0		
	O	0.5238	0.4762	0.0053	1.0		
**NPM2**	O	0.0000	0.0000	0.0000	1.0	a=b=2.97 c=9.99	R_P =83.6 R_{wp} =51.3 R_{exp}=50.1 χ^2 = 1.05
	Fe	0.3333	0.6667	0.337	1.0		
	Fe	0.3333	0.6667	0.663	1.0		
	Fe	0.3333	0.666	0.837	1.0	α = β = 90° γ=120°	
	Fe	0.3333	0.6667	0.163	1.0		
	O	0.6667	0.3333	0.500	1.0		
**NPM3**	O	0	0	0	1.0	a=b=2.97 c=9.36	R_P =93.1 R_{wp} =64.3 R_{exp}=60.9 χ^2 = 1.13
	Fe	0.3333	0.6667	0.337	1.0		
	Fe	0.3333	0.6667	0.663	1.0		
	Fe	0.3333	0.6667	0.837	1.0	α = β = 90° γ=120°	
	Fe	0.3333	0.6667	0.163	1.0		
	O	0.6667	0.3333	0.500	1.0		
**NPM4**	Fe-tet	0.125	0.125	0.125	1.0	a=b=c=8.36 α = β = γ = 90°	R_P =43.1 R_{wp} =22.3 R_{exp}=20.8 χ^2 = 1.15
	Fe-oct	0.500	0.500	0.500	1.0		
	O	0.250	0.250	0.250	1.0		
**NPM5**	Fe-tet	0.125	0.125	0.125	1.0	a=b=c=8.36 α = β = γ= 90°	R_P =31.7 R_{wp} =15.8 R_{exp}=15.5 χ^2 = 1.05
	Fe-oct	0.500	0.500	0.500	0.9		
	O	0.250	0.250	0.250	0.9		
**NPM6**	Fe-tet	0.125	0.125	0.125	1.0	a=b=c=8.36 α = β = γ=90°	R_P =37.4 R_{wp} =18.7 R_{exp}=17.8 χ^2 = 1.10
	Fe-oct	0.500	0.500	0.500	1.0		
	O	0.250	0.250	0.250	1.0		

Table S2
Crystallite size and Caglioti parameters.

sample	Crystalite size (nm)	U	V	W	X	Y
**NPM1**	5	0.083	-0.027	0.066	2.142	1.218
**NPM2**	3	0.968	-0.203	0.891	0.176	1.857
**NPM3**	3	0.010	-0.026	0.015	2.126	1.941
**NPM4**	6	0.026	-0.030	0.027	0.188	0.996
**NPM5**	10	0.061	-0.184	0.080	0.000	0.555
**NPM6**	10	0.303	-0.364	0.108	0.009	0.542

Table S3
Statistical parameters for NPM1-6 samples obtained from the PSD histogram. Dm: diameter of particle, SD: standard deviation, PDI: polydispersity index.

sample	Dm (SD) (nm)	PDI
NPM1	4 (1)	0.32
NPM2	4 (1)	0.35
NPM3	4 (1)	0.34
NPM4	11 (1)	0.01
NPM5	12 (1)	0.02
NPM6	11 (1)	0.01

Table S4

Elemental Characterization by XPS: Bond Energy Profiles and Surface Composition of NPM6.

Sample			Pos. (eV)	FMHW (eV)	% area
		-C	284.5	1.7	61.8
	C 1s	-CO	285.7	2.2	27.5
		-COO	288.6	1.3	10.7
		Fe^{2+}	709.7	1.7	21.6
		Fe^{3+}	710.5	2.0	36.6
**NPM6**	Fe 2p	Fe^{3+}	711.8	2.0	19.3
		Fe^{2+} sat.	713.4	3.2	13.0
		Fe^{3+} sat.	718.9	3.6	9.5
		O-Fe	529.4	1.3	44.2
	O 1s	O-C	530.7	1.8	34.1
		O-H	532.3	2.1	21.7

Table S5

Elemental composition of the elements found in the NPM6 sample by XPS.

	Composition (%at)						
Sample	Fe	O	N	C	Cl	S	Si
NPM6	9.1	34.3	0.3	54.6	0.0	0.0	1.7

Table S6: Magnetic parameters obtained for the NPM1-6 samples. M_r is the remanent magnetization, H_c is the coercive field, χ is the paramagnetic susceptibility, and K_{eff} is the effective anisotropy constant.

**Sample**	***T*** **(K)**	M_r **(emu g^{-1}) ±0.1**	H_c **(Oe) ±3**	M_s **(emu g^{-1}) ±1**	M_r/M_s	**χ 10^{-5} (emu/g kOe) ±0.1**	$K_{eff} \times 10^5$ **(J m^{-3}) ±0.1**
**NPM1**	4	4.0	1248	12	0.3	9.9	1.9
	300	0.1	24	6	0.02	7.4	0.9
**NPM2**	4	2.0	2190	7	0.3	8.4	1.3
	300	0.02(1)	20	2	0.01	5.9	0.4
**NPM3**	4	2.0	2110	7	0.2	8.4	1.4
	300	0.001(1)	5	1	0.001	6.1	0.3
**NPM4**	4	14.0	251	56	0.3	2.9	3.0
	300	1.0	16	47	0.02	3.4	2.7
**NPM5**	4	18.0	262	77	0.2	2.6	4.0
	300	0.8	9	69	0.01	2.3	3.0
**NPM6**	4	14.0	220	55	0.3	2.4	3.1
	300	2.0	23	46	0.04	2.7	2.6

Table S7

Hyperfine parameters for the NPM6 sample. RAA: relative spectral absorption area, δ: isomer shift vs Fe at 300 K; B_{hf}: Magnetic hyperfine field; QS: Quadrupole splitting (fixed); σ: width of Gaussian distribution of B_{hf}; W: Lorentzian width (fixed=0.24 mm s^{-1}).

Samples	Components	RAA (%)	δ vs. Fe 300 K (mm s^{-1})	B_{hf} (T)	QS (mm s^{-1})	Γ (mm s^{-1})
**NPM6**	MFD	92	0.29	36.8	0.0	0.35
	Doublet	8	0.29	0	0.6	0.32
**error**		±3	±0.02	±0.5	±0.02	±0.03

Table S8

Hyperfine parameters for the NPM6 sample. RAA: relative spectral absorption area, δ: isomer shift vs Fe at 15 K; B_{hf}: Magnetic hyperfine field; Q: Quadrupole splitting (fixed); σ: width of Gaussian distribution of B_{hf}; W: Lorentzian width (fixed =0.24 mm s^{-1}).

Samples	Components	RAA (%)	δ vs. Fe 300 K (mm/s)	B_{hf} (T)	Q (mm/s)	Γ (mm/s)
**NPM6**	Site A	37	0.28	50.1	0	0.70
	Site B	63	0.48	52.7	0	0.70
**error**		±3	±0.02	±0.5	±0.02	±0.03

Table S9

Elemental composition of the elements found in the after-adsorption samples by XPS.

	Composition (% at)						
Sample	Fe	O	N	C	Cl	S	Si
MB2	10.6	35.4	0.5	52.1	0.0	0.0	1.4
MB7	9.9	37.8	0.5	50.0	0.0	0.0	1.7
MG2	11.5	35.2	0.4	51.6	0.0	0.0	1.3
MG7	12.1	40.9	0.4	45.3	0.0	0.0	1.3

Supplementary Figures

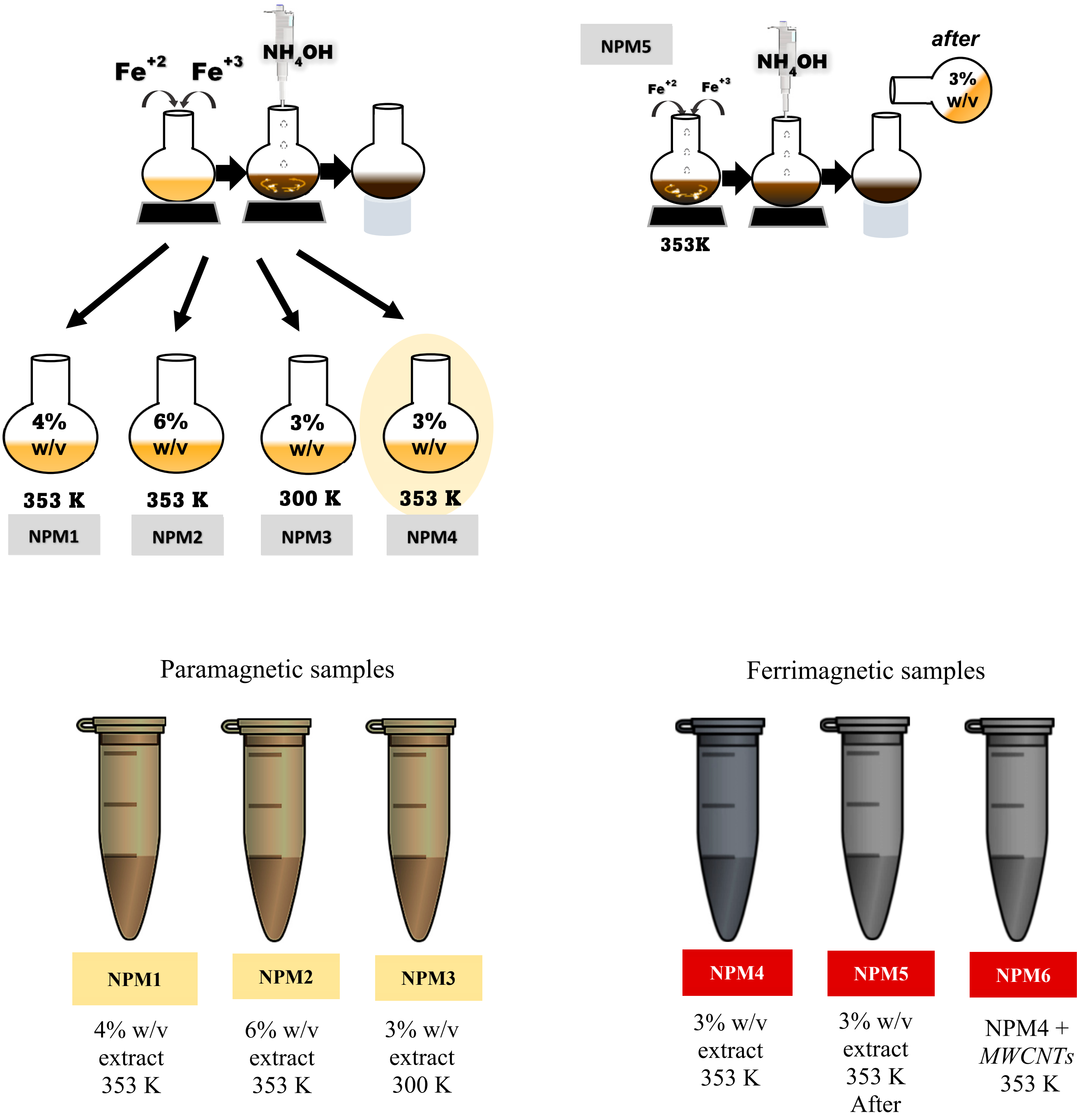

Scheme S1. (a) Synthesis of iron oxide NPs assisted with *Citrus reticulata* peel extract. (b) NPM1-6 synthesized samples and classified into paramagnetic: NPM1, NPM2, and NPM3; and (c) Ferrimagnetic: NPM4, NPM5, and NPM6.

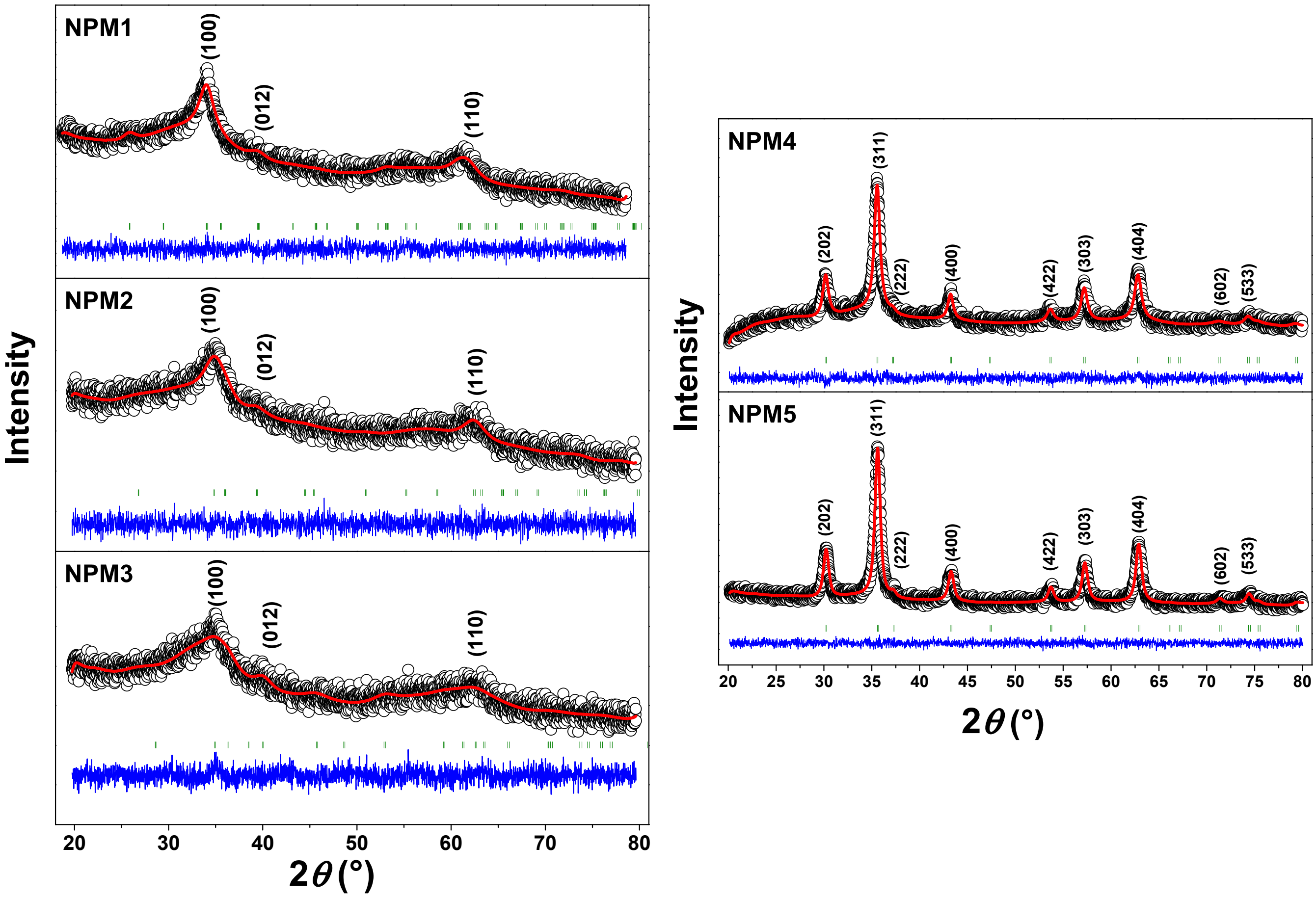

Figure S1. Rietveld refinement of the NPM1-5 samples. In the graphical representation, the black circles correspond to the experimental data, the red lines indicate the calculated diffractogram, the blue lines represent the difference or residual, and the green lines indicate the Bragg reflection positions.

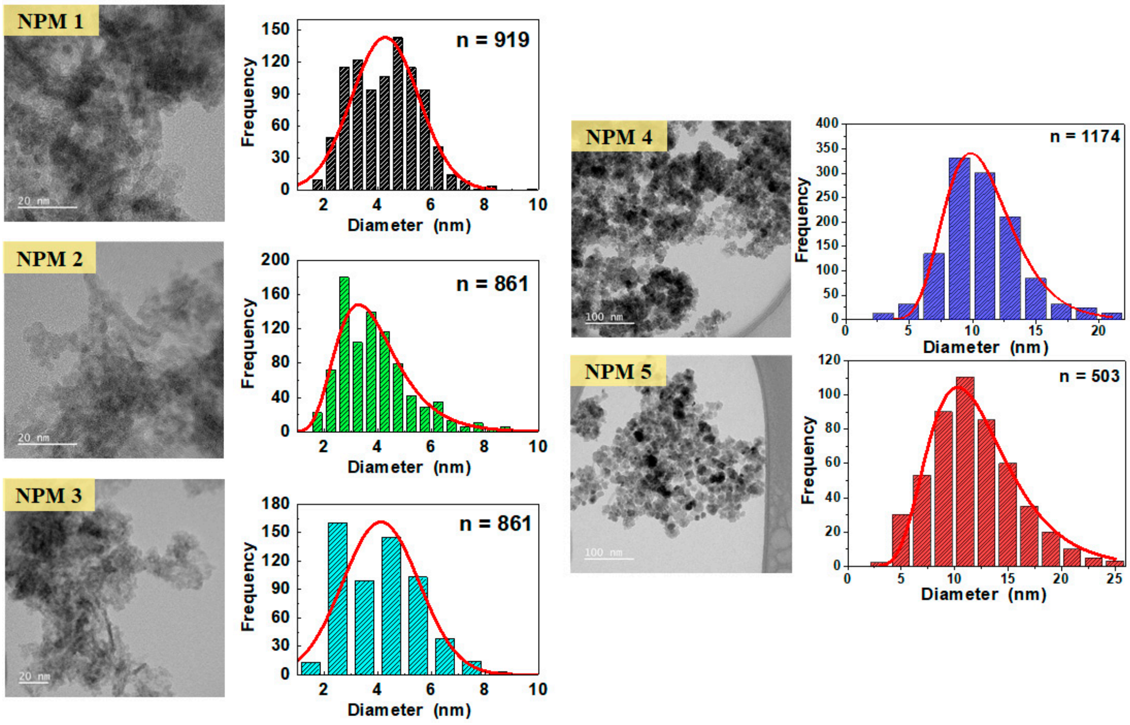

Figure S2. TEM images and size distribution of the synthesized NPM1-5 samples. n indicates the total counted particles to obtain the PSD histogram.

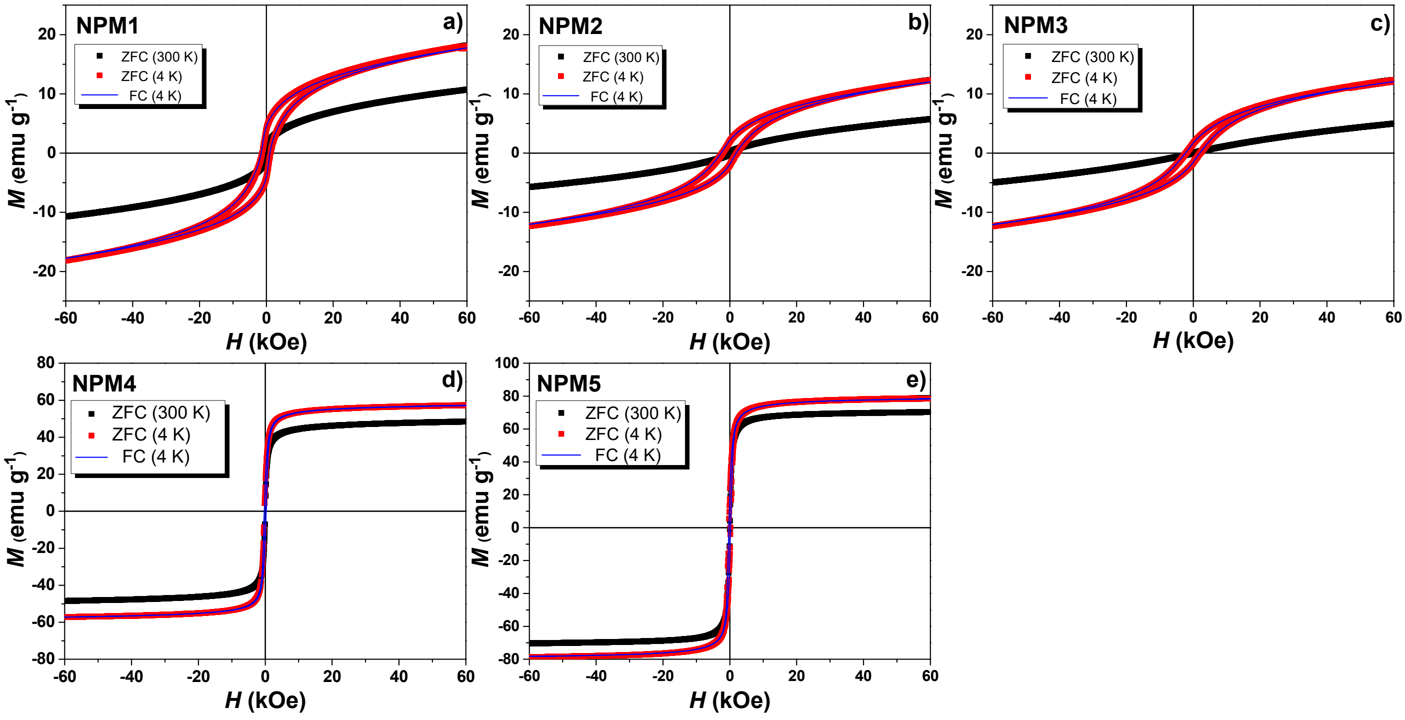

Figure S3. ZFC and FC $M(H)$ curves for NPM1-5 samples at 300 K and 4 K. H_{FC} = 1 kOe. (a-c) paramagnetic and (d-e) ferrimagnetic samples.

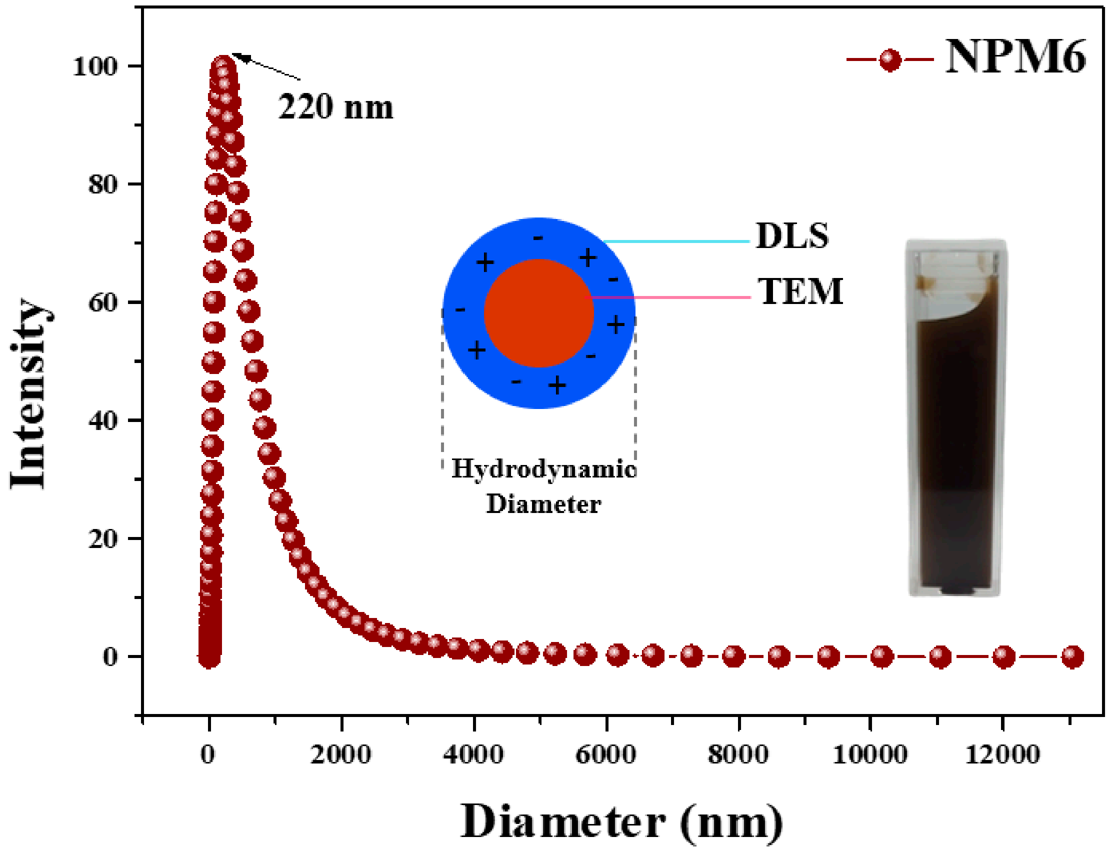

Figure S4. Lognormal distribution of hydrodynamic diameter of NPM6.

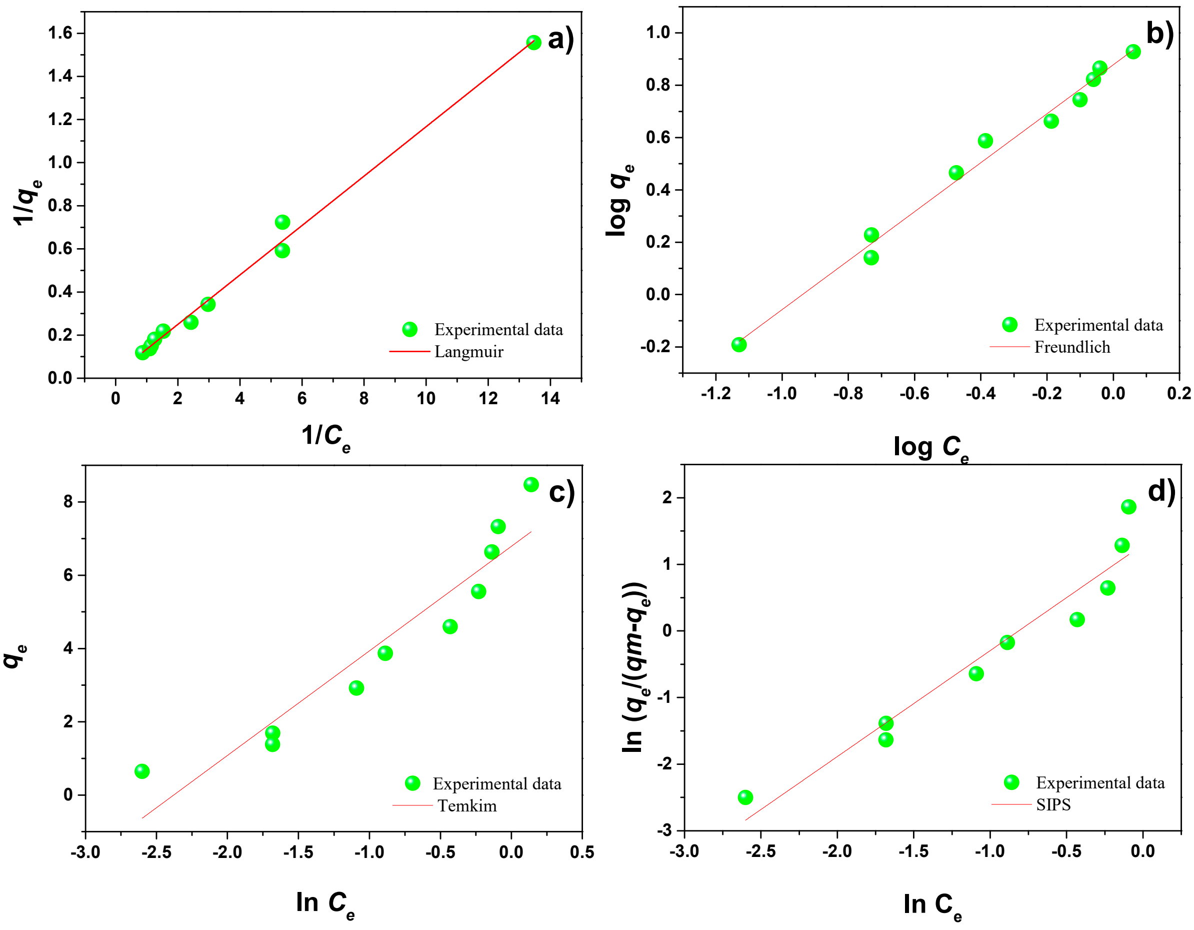

Figure S5. Linear fits of adsorption isotherm for the MG dye.

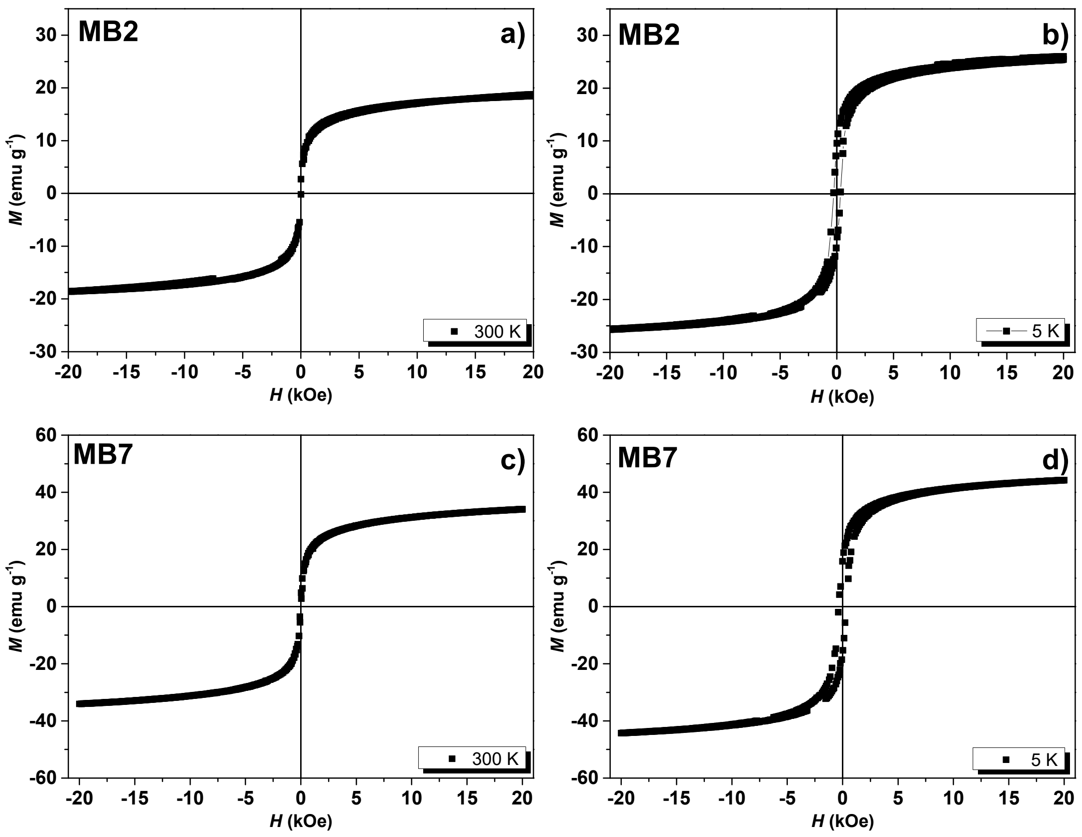

Figure S6. $M(H)$ curves of NPM6 recovered in the second (2nd) and seventh (7th) use cycles of MB (a) at 300 K after the 2nd use, (b) at 5 K after the 2nd use, (c) at 300 K after the 7th use, and (d) at 5 K after the 7th use.

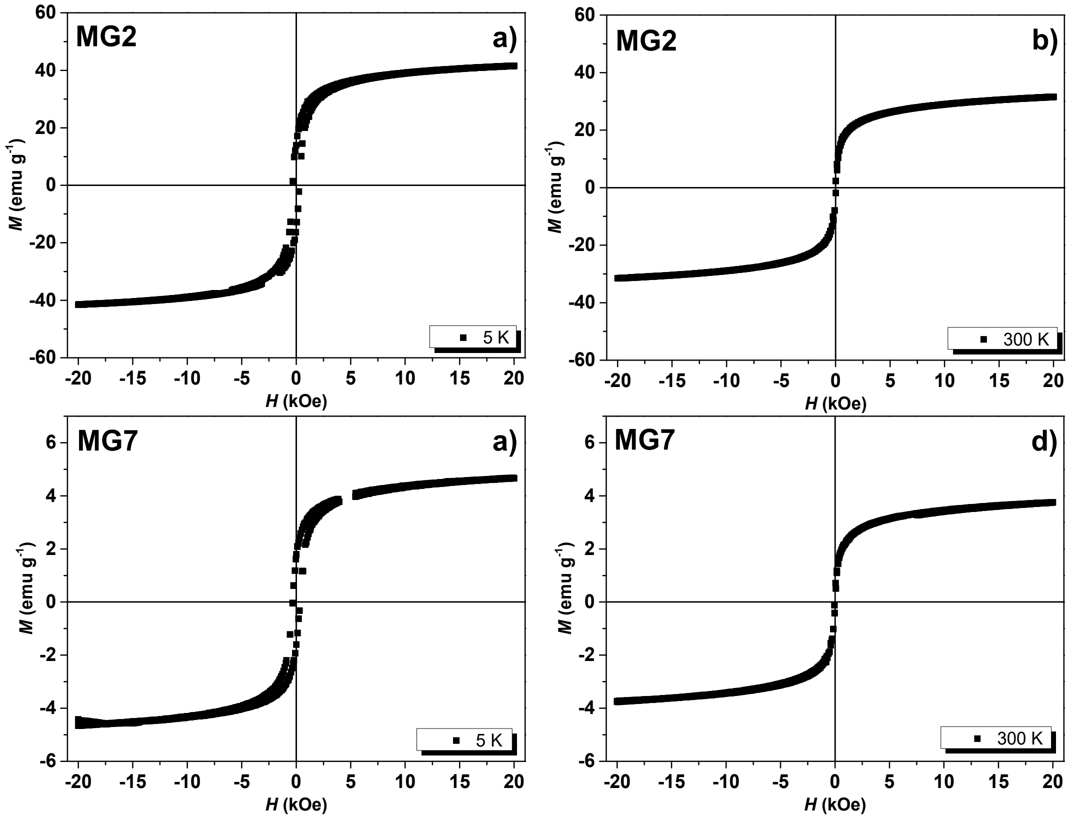

Figure S7. $M(H)$ curves of NPM6 recovered in the second (2nd) and seventh (7th) use cycles of MG (a) at 300 K after the 2nd use, (b) at 5 K after the 2nd use, (c) at 300 K after the 7th use, and (d) at 5 K after the 7th use.
